# Intercontinental insights into autism spectrum disorder: a synthesis of environmental influences and DNA methylation

**DOI:** 10.1093/eep/dvae023

**Published:** 2024-11-07

**Authors:** George E Kuodza, Ray Kawai, Janine M LaSalle

**Affiliations:** Department of Medical Microbiology and Immunology, Perinatal Origins of Disparities Center, MIND Institute, Genome Center, Environmental Health Sciences Center, University of California Davis, Davis, CA 95616, United States; Department of Medical Microbiology and Immunology, Perinatal Origins of Disparities Center, MIND Institute, Genome Center, Environmental Health Sciences Center, University of California Davis, Davis, CA 95616, United States; Department of Medical Microbiology and Immunology, Perinatal Origins of Disparities Center, MIND Institute, Genome Center, Environmental Health Sciences Center, University of California Davis, Davis, CA 95616, United States

**Keywords:** autism spectrum disorders, DNA methylation, environmental factors, prevalence

## Abstract

Autism Spectrum Disorder (ASD) is a complex neurodevelopmental disorder characterized by a broad range of symptoms. The etiology of ASD is thought to involve complex gene–environment interactions, which are crucial to understanding its various causes and symptoms. DNA methylation is an epigenetic mechanism that potentially links genetic predispositions to environmental factors in the development of ASD. This review provides a global perspective on ASD, focusing on how DNA methylation studies may reveal gene–environment interactions characteristic of specific geographical regions. It delves into the role of DNA methylation in influencing the causes and prevalence of ASD in regions where environmental influences vary significantly. We also address potential explanations for the high ASD prevalence in North America, considering lifestyle factors, environmental toxins, and diagnostic considerations. Asian and European studies offer insights into endocrine-disrupting compounds, persistent organic pollutants, maternal smoking, and their associations with DNA methylation alterations in ASD. In areas with limited data on DNA methylation and ASD, such as Africa, Oceania, and South America, we discuss prevalent environmental factors based on epidemiological studies. Additionally, the review integrates global and country-specific prevalence data from various studies, providing a comprehensive picture of the variables influencing ASD diagnoses over region and year of assessment. This prevalence data, coupled with regional environmental variables and DNA methylation studies, provides a perspective on the complexities of ASD research. Integrating global prevalence data, we underscore the need for a comprehensive global understanding of ASD’s complex etiology. Expanded research into epigenetic mechanisms of ASD is needed, particularly in underrepresented populations and locations, to enhance biomarker development for diagnosis and intervention strategies for ASD that reflect the varied environmental and genetic landscapes worldwide.

## Introduction

Autism spectrum disorder (ASD) is a category of neurodevelopmental disorders defined by deficits in both social communication and language, combined with repetitive and restrictive behaviors. A significant challenge in studying the etiology of ASD is the change in diagnostic criteria over time, making it difficult to determine whether there is an actual increase in the incidence of ASD versus improved diagnosis [1]. While a diagnosis of ASD has become more standardized in recent years, there are still significant disparities that exist by child gender, access to health care, and parental education within countries such as the USA [2, 3]. Globally, disparities in ASD diagnosis are even more apparent, making it currently unfeasible to come up with an accurate estimate of ASD prevalence worldwide [4].

The lack of reliable ASD diagnosis also limits the inclusion of diverse populations in genetic and environmental studies. The etiology of ASD is complex, involving both genetic and environmental contributors to risk. While there has been much success in identifying rare genetic causes of ASD, any single gene can only explain <1% of total ASD cases individually and only <10% collectively [5, 6]. While ASD is considered one of the most heritable neuropsychiatric disorders based on monozygotic versus dizygotic twin studies, the heritability estimates have varied widely by size and year of the study, as well as geographic and demographic differences [7–10]. Familial risk for ASD appears to be more consistent across “baby sib” studies, where the risk of having a second child with ASD is 15–17 times higher than the general population [11–13]. Common genetic studies for ASD have been mostly limited to US and European researchers studying predominantly white ASD cases from highly educated parents. For example, the largest ASD genome-wide association study (GWAS) identified only five loci at genome-wide significance [14]. There was a strong overlap with GWAS of educational attainment and a positive correlation with cognitive tests [15], despite the opposite being expected based on cognitive tests in ASD cases [16]. Polygenic risk scores also have limited effect sizes that are generally below those for the more common medical and environmental risk factors for ASD, including maternal obesity, preterm birth, or valproate use [17, 18]. However, it is important to note that as GWAS sample sizes increase and include more diverse participants, polygenic risk scores for ASD will likely improve, potentially explaining a more significant proportion of variance in ASD phenotypes.

Therefore, ASD is currently lacking reliable molecular tests and biomarkers that can assess the risk for ASD diagnosis, which is usually between the ages of 3 and 5 years worldwide [19]. Some studies have demonstrated the effectiveness of early behavioral interventions that can improve the developmental trajectory of toddlers showing early signs of ASD [20, 21]. DNA methylation is an epigenetic modification throughout the genome that can vary according to genetic, environmental, and gene × environmental (G×E) factors [22, 23]. Unlike transcription, DNA methylation patterns are “metastable,” meaning they can be stable for long periods across the lifespan and changeable under the right conditions. DNA methylation “signatures” of ASD refer to combined groups of DNA methylation changes that have been identified in the brain as well as a variety of surrogate tissues collected both before (placenta, cord blood, newborn blood) or after (blood, saliva, buccal) diagnosis of ASD [24]. DNA methylation patterns are at the interface of genetic and environmental interactions. This was well demonstrated in a study by Czamara *et al*., which found that among various neuropsychiatric conditions, ASD showed the greatest enrichment of genetic loci identified through GWAS, which were also associated with DNA methylation changes [23]. These changes were best explained by a G×E model, highlighting the significant role that both genetic predisposition and environmental factors play in ASD.

**Figure 1.**
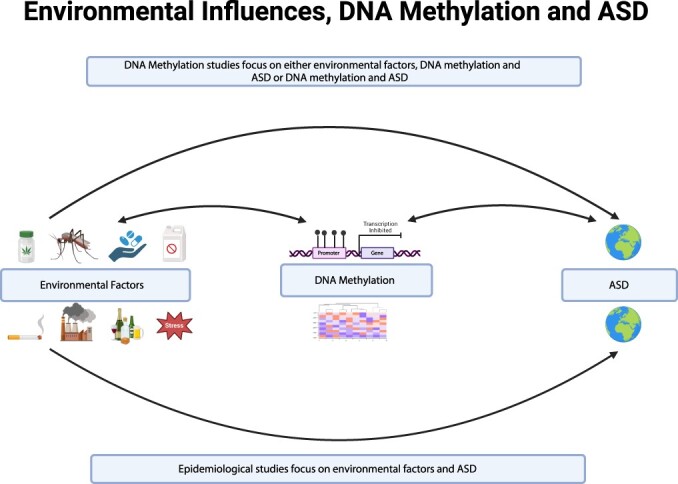
This review summarizes molecular studies investigating the possible relationship between environmental factors and ASD, with DNA methylation as a direct association. For continents with an underrepresentation of DNA methylation studies, we also include epidemiological studies that directly examined associations between environmental factors and ASD. We have also included ASD prevalence estimates for the countries covered in the review. Figure made using Biorender.

**Figure 2.**
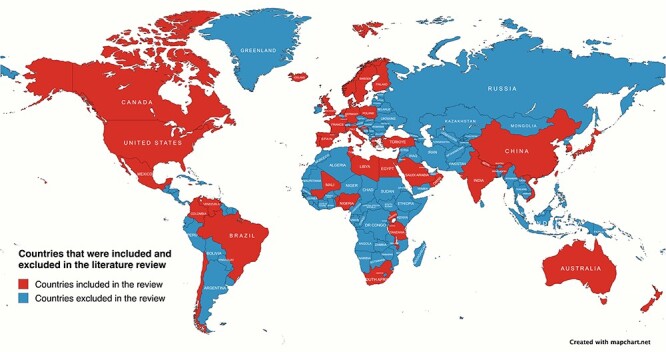
This map shows the countries included (red) or excluded (blue) in the literature review based on available published studies. The countries included in the study were Australia, Bangladesh, Brazil, Canada, Chile, China, Colombia, Denmark, Ecuador, Egypt, England, Finland, France, Germany, Greece, Iceland, India, Israel, Italy, Japan, Lebanon, Libya, Mali, Mexico, Nepal, New Zealand, Nigeria, Norway, Oman, Poland, Portugal, Qatar, Saudi Arabia, South Africa, South Korea, Spain, Sweden, Taiwan, Tunisia, Uganda, USA, Venezuela, and Vietnam.

The main objective of this review is to take a global perspective on ASD and consider the importance of early detection and intervention, with the goal that every child may reach their full potential. Globally, populations differ by genetics and environmental exposures, so it is essential not to assume that results from research performed in North America or Europe will apply to other geographic locations. We, therefore, will discuss research studies investigating the connections between ASD and environmental exposures, as well as those using DNA methylation signatures or candidate biomarkers as direct associations (Fig. 1). Table 1 lists and summarizes these studies, ordered by continents, with the most studies investigating DNA methylation and environmental exposures in ASD. For continents with fewer DNA methylation studies, we include those investigating only environmental associations with ASD or neurodevelopmental disorders more generally. We will further attempt to summarize ASD prevalence data for the continents and countries where these data have been published. Table 2 provides a comprehensive summary of the studies included in the prevalence section. Figure 2 shows the geographical distribution of countries included in the study for their contributions to ASD research based on published studies of either molecular or epidemiological research.

**Table 1.** Key findings on the association between environmental exposures, DNA methylation, and autism spectrum disorder risk, including additional studies

Study	Country	Environmental exposure	Biological specimen	Sample size	ASD mean/ range age	ASD/Neurodevelopment diagnostic tool	Techniques for DNA methylation analysis	Approach to the analyzed genomic region	Results environment + DNA methylation + ASD/neurodevelopment link or DNA methylation + ASD/neurodevelopment link or environment + ASD/neurodevelsopment link
North America									
Schmidt *et al*. [68]	United States	Prenatal exposures	Placenta	24 ASD, 23 TD	Diagnosed ASD at 36 months	DSM-IV| DSM-V| ADOS	WGBS	Genome wide	Strong association between professional outdoor pesticide use and altered placental DNA methylation in PMDs
Ladd-Acosta *et al*. [69]	United States	NO_2_ and O_3_	Cord blood and placenta	Mother–child pairs (*n* = 192 children)	2–12 years	ADOS| ADI-R| SRS| M-CHAT| MSEL| VABS	Illumina Infinium, HumanMethylation450K BeadChip (Illumina, San Diego, CA)	Genome-wide RR	Neonates exposed to prenatal O_3_ had methylation loss, affecting genes *RNF39, CYP2E1, PM20D1* (cord blood), *ZNF442, PTPRH, SLC25A44, F11R, STK38* (placenta), with female-specific NO_2_-related variations in *CYP2E1*
Aung *et al*. [73]	United States	Heavy metals (lead, cadmium, manganese)	Peripheral blood	97 women with previous ASD births	N/A	N/A	Illumina Infinium, HumanMethylation450K BeadChip (Illumina, San Diego, CA)	Genome-wide RR	Results showed hypermethylation at 11 sites associated with lead, near genes *CYP24A1, ASCL2, FAT1, SNX31, NKX6-2, LRC4C, BMP7, HOXC11, PCDH7, ZSCAN18, and VIPR2*. Manganese was associated with hypermethylation near *ARID2*. Effect estimates for DNA methylation sites associated with cadmium, lead, and manganese were highly correlated.
Mouat *et al*. [70]	United States	PCB	Peripheral blood	*n* = 95	Around 36 months in age	ADOS| ADI-R| MSEL	WGBS	Genome-wide	PCBs prevalent in pregnant women’s serum, influenced by maternal age. Modules linked PCBs to infant neurodevelopment, with *CSMD1 and AUTS2* genes connecting maternal PCB levels to child neurodevelopment outcomes
Mitchell *et al*. [56]	United States	PBDE, PCB	Brain tissue	32 ASD, 43 TD, 4 Angelman, 4 Down Syndrome,6 proximal 15q duplication,6 Prader-Willi syndrome,12 Rett Syndrome	4-60 years	N/A	Pyrosequencing	Genome-wide RR	Controls and non-idiopathic ASD individuals had lower PCB 95 levels than those with maternal 15q11-q13 duplication or Prader-Willi syndrome, with Dup15q predicting lower repetitive DNA methylation
Feinberg *et al*. [80]	United States	Prenatal exposures	Semen	Paternal sample = 45, offspring sample = 31	Paternal= 36.96 years, child = 3 years	SRS	CHARM; Illumina Infinium, HumanMethylation450K BeadChip (Illumina, San Diego, CA) was also used at different study locations.	Genome-wide RR	Only paternal education linked to child SRS scores. Overlap in child and paternal SRS-associated DMRs found in genes *WWOX, SALL3, AJAP1, TGM3, Iroquois Homeobox 4*
Schrott *et al*. [82]	United States	THC	Semen	24 males	18–40 years	N/A	RRBS, Pyrosequencing	Candidate gene*(DLGAP2)*	By using bisulfite pyrosequencing on nine clustered CpG sites, they discovered hypomethylation linked to cannabis use
Schrott *et al*. [81]	United States	THC	Semen	6 vehicle controls and 6 replicates	N/A	N/A	Pyrosequencing	Candidate gene *(BRD4, CADPS, EXOC3, FBXO40, MED12 L, TSPAN17, CMPK2, GRIK2, KLF16, HCN1, NR4A2)*	Cannabis significantly alters methylation in spermatogenic cells, affecting ASD candidate genes *HCN1, NR4A2*, and imprinted genes *SGCE, GRB10, PEG3*, impacting spermatogenic stem and spermatid-like cells
Siu *et al*. [86]	Canada	N/A	Peripheral blood	52 ASD,9 16p11.2 deletions, 7 CHD8±	10.45 ± 3.306 years	N/A	Illumina Infinium, HumanMethylation450K BeadChip (Illumina, San Diego, CA)	Genome-wide RR	DNA methylation signatures in 16p11.2del and CHD8± subgroups differentiate individuals from each other and from controls and idiopathic ASD cases with high sensitivity and specificity
Siu *et al*. [85]	Canada	N/A	Peripheral blood (ASD), saliva (ADHD, OCD)	248 ASD, 151 TD blood samples, 38 ADHD, 38 OCD, and 65 TD saliva samples	4–12 years	ADOS| ADI-R	Pyrosequencing	Candidate gene *(OXTR)*	People with ASD or ADHD exhibited extreme DNA methylation at specific sites, correlating with higher CBCL social problems scores in ADHD and lower IQs in ASD
Mordaunt *et al*. [184]	United States	N/A	Cord blood	76 ASD 76 TD	N/A	ADOS| MSEL| DSM-V	WGBS	Genome-wide	Sex-specific DMRs in cord blood differentiate ASD, with significant enrichment in brain expression, X chromosome, and ASD epigenetics. Autosomal DMRs favor promoter/bivalent states; X-linked DMRs show sex differences
Zhubi *et al*. [58]	United States	N/A	Brain tissue	8 ASD 10 TD	30 ± 4 years	N/A	Methyl and hydroxymethyl DNA immunoprecipitation	Genome-wide RR	Significant mRNA reductions in *RELN, GAD1*, increases in TET1, 2, 3 enzymes, with *MECP2, DNMT1* binding increases at *GAD1, RELN, GAD2* promoters, affecting 5mC levels, minimal 5hmC changes
L. Zhu *et al*. [55]	United States	N/A	Brain tissue	54 ASD 43 TD	N/A	N/A	PCR	Candidate gene *(SHANK3*)	Increased DNA methylation in intragenic CGIs (CGI-2, CGI-3, and CGI-4) in ASD brain tissues was observed, leading to altered expression and alternative splicing of *SHANK3* isoforms
Wong *et al*. [57]	United States	N/A	Brain tissue	43 ASD 38 TD	N/A	N/A	Illumina Infinium, HumanMethylation450K BeadChip (Illumina, San Diego, CA)	Genome-wide RR	Widespread DNA methylation differences in idiopathic ASD and dup15q ASD subtype, with shared and distinct patterns, highlighting immune, synaptic, and neuronal regulation genes in cortical co-methylation networks
Nardone *et al*. [185]	United States	N/A	Brain tissue	16 ASD 15 TD	N/A	N/A	Illumina Infinium, HumanMethylation450K BeadChip (Illumina, San Diego, CA)	Genome-wide RR	Identified 58 ASD-related DMRs in *ABAT, GABBR1*, and MicroRNAs genes, with co-methylation modules linked to neuronal, GABAergic, immune genes, and overlap with neurodevelopment-associated DMRs
Nardone *et al*. [54]	United States	N/A	Brain tissue	13 ASD 12 TD	N/A	N/A	Illumina Infinium, HumanMethylation450K BeadChip (Illumina, San Diego, CA)	Genome-wide RR	Dysregulated CpGs in ASD cortical regions BA10 and BA24 affect immune genes (*C1Q, C3, ITGB2, TNF-α, IRF8, SPI1*) and synaptic genes, inversely correlating with expression, including *HDAC4 and C11orf21/TSPAN32*
Ladd-Acosta *et al*. [53]	United States	N/A	Brain tissue	20 ASD 21 TD	N/A	ADI-R| ADOS	Illumina Infinium, HumanMethylation450K BeadChip (Illumina, San Diego, CA)	Genome-wide RR	Identified significant DMRs in *PRRT1, TSPAN32, C11orf21, ZFP57, and SDHAP3* across brain regions, suggesting regulatory roles in ASD
Corley *et al*. [59]	United States	N/A	Brain tissue	17 ASD 17 TD	N/A	N/A	Illumina Infinium, HumanMethylation450K BeadChip (Illumina, San Diego, CA)	Genome-wide RR	Genome-wide methylome analyses showed alterations in neurodevelopment genes, targeting intragenic and bivalently chromatin domains, linked to abnormal mRNA splicing events in ASD-relevant genes
Vogel Ciernia *et al*. [60]	United States	N/A	Brain tissue	17 ASD 10 TD 12 Rett Syndrome 5 Dup15q Syndrome	N/A	N/A	WGBS	Genome wide	Methylation changes in NDDs showed unique DMRs with shared functions in neurons, microglia, enriched in promoter regions, and sensitive transcription factor binding sites, indicating common regulatory mechanisms
James *et al*. [61]	United States	N/A	Brain tissue	13 ASD 13 TD	15.5 ± 9.5 years	DSM-IV	Hydroxymethylated DNA immunoprecipitation (hMeDIP)	Candidate gene(*EN2*)	Autism cerebellum shows increased 5-hmC content, *DNMT3A* and *DNMT3B* expression, *TET1* and *TET3* translocase genes, and 8-oxo-dG content, with a positive correlation with *EN-2* gene expression
Bakulski *et al*. [186]	United States	Prenatal exposures	Peripheral blood, cord blood, and placenta	232 mothers participated in the study	21–44 years	DSM-V| SRS| MSEL| VABS.	Illumina Infinium, HumanMethylation450K BeadChip (Illumina, San Diego, CA)	Genome-wide RR	No single methylation site linked to ASD at genome-wide significance, but sites nominally associated with ASD were highly enriched for SFARI genes across multiple tissues, overlapping at 144 SFARI genes
Andrews *et al*. [187]	United States	N/A	Cord blood, peripheral blood, and fetal brain	Fetal brain, 166; peripheral, 339; blood cord blood, 121; lung-210	N/A	N/A	Illumina Infinium, HumanMethylation450K BeadChip (Illumina, San Diego, CA)	Genome-wide RR	The study identifies SNPs in cord and peripheral blood linked to DNA methylation in ASD, showing enrichment in fetal brain and blood meQTLs, implicating immune-related pathways
V. W. Hu *et al*. [188]	United States	N/A	Lymphoblastoid cell lines	21 ASD 21 TD siblings	8.45 years	ADI-R	Affymetrix Human Promoter 1.0 R GeneChips	Genome-wide RR	Significant DNA methylation differences between idiopathic ASD individuals and unaffected siblings, enriched in autism susceptibility genes and common ASD pathways, highlighting genetic underpinnings of the disorder
Bahado-Singh *et al*. [117]	United States	N/A	Neonatal dried blood spots	14 ASD and 10 TD	At birth (29 h–79 h) after birth	DSM-IV	Illumina Infinium, HumanMethylation450K BeadChip (Illumina, San Diego, CA)	Genome-wide RR	CpG methylation changes were found in 230 loci, associated with 249 genes, including some previously associated with ASD (*EIF4E, FYN, SHANK1, and VIM*). The best predictive CpG sites were associated with seven genes: *NAV2, OXCT1, LOC389033, MYL9, ALS2CR4, C19orf73, and ASCL2*
Andrews *et al*. [189]	United States	N/A	Peripheral blood	453 ASD and 515 TD	3–5 years	ADOS| ADI-R	Illumina Infinium, HumanMethylation450K BeadChip (Illumina, San Diego, CA)	Genome-wide RR	No CpG sites reached EWAS threshold significance. Most DMPs were associated with the *CENPM, FENDRR, SNRNP200, PGLYRP4, EZH1, DIO3*, and *CCDC181* genes
Jasoliya *et al*. [190]	United States	N/A	Peripheral blood	23 ASD, 23 FXS with ASD, 11 TD.	2–6 years	ADOS| DSM-V	Illumina Infinium, HumanMethylation BeadChip (Illumina, San Diego, CA)	Genome-wide RR	Study found differentially methylated sites and regions, with genes *PAK2, GTF2I, and FOXP1* from the SFARI database, highlighting their importance in brain development among ASD groups
Bahado-Singh *et al*. [64]	United States	N/A	Placenta	14 term autism cases (7 males, 7 females)	N/A	DSM-IV	Illumina Infinium, HumanMethylation450K BeadChip (Illumina, San Diego, CA)	Genome-wide RR	Study found 9655 autism-related differentially methylated CpGs, both intergenic and intragenic. AI analysis showed high accuracy for autism detection. Deep learning yielded an AUC (95% CI) of 1.00 (1.00–1.00) for autism detection using intra- or intergenic markers by themselves or combined
Y. Zhu *et al*. [66]	United States	N/A	Placenta	83 ASD 108 TD 13 Non-TD	N/A	ADOS| ADI-R| MSEL	WGBS	Genome-wide	Study identified NHIP as a novel ASD risk gene, with decreased expression in ASD placenta/brain. Overexpression affects cellular proliferation and gene expression, intersecting with established ASD risk genes
Y. Zhu *et al*. [65]	United States	N/A	Placenta	20 ASD 21 TD	N/A	ADOS| ADI-R| DSM-V| MSEL	WGBS	Genome-wide	400 DMRs in ASD placentas, enriched at promoters and, mapped to 596 genes functionally enriched in neuronal development, and overlapped genetic ASD risk. ASD DMRs *at CYP2E1* and *IRS2* reached genome-wide significance. Methylation at *CYP2E1* associated with both ASD diagnosis and genotype within the DMR In contrast, methylation at *IRS2* was unaffected by within DMR genotype but modified by periconceptional maternal prenatal vitamin use
Schroeder *et al*. [67]	United States	N/A	Placenta	24 ASD and 23 TD	N/A	DSM-IV| DSM-V| ADOS	WGBS	Genome-wide	The study found stable PMD and HMD locations in placental methylomes but greater individual variability in PMD methylation. An HMD near *DLL1*, linked to ASD, showed higher methylation validated by pyrosequencing
Lintas *et al*. [62]	United States	N/A	Brain tissue	6 ASD 6 TD	21± 2.9 years	N/A	Bisulfite treatment, PCR (real time), *in silico* analysis	Candidate gene(*RELN*)	ASD patients have higher methylated CpG islands and heavier methylation in the 5′ region of the *RELN* gene promoter, compared to controls. Three distinct methylation patterns are discernible, each correlated with different reductions in reelin gene expression in ASD individuals compared to controls
Andari *et al*. [191]	United States	N/A	Saliva	35 ASD 64 TD	27.02 ± 5.34 years	DSM-V| ADOS| ADI-R| SRS	EpiTYPER on the MassARRAY system by Agena Bioscience	Candidate gene(*OXTR)*	Adults with ASD have higher *OXTR* methylation in intron 1, linked to clinical symptoms and cortico-cortical hypoconnectivity. Exon 1 CpG site methylation correlates with social deficits and striatal-cortical hyperconnectivity
Feinberg *et al*. [78]	United States	N/A	Semen	44 Fathers of ASD	N/A	N/A	Illumina Infinium, HumanMethylation450K BeadChip (Illumina, San Diego, CA)	Genome-wide RR	Identified 193 sperm DMRs linked to offspring AOSI scores, clustering near developmental genes and the *SNORD* family within the Prader-Willi syndrome gene cluster, indicating hereditary ASD risk factors
Europe									
Maggio *et al*. [79]	Denmark	DDE, DDT, seafood neurotoxicants (persistent organic pollutants)	Semen	52 semen samples	N/A	N/A	WGBS	genome wide	The study suggests sperm DNA methylation is affected by exposure to DDE and other persistent environmental contaminants, highlighted in the Faroese cohort with high EDC levels from consuming whale products
Hannon *et al*. [90]	Denmark	Smoking in pregnancy	Neonatal dried blood spot	629 ASD cases and 634 TD	Mean gestational age of 39.6 weeks (SD = 1.77 weeks)	ICD-10	Illumina Infinium, HumanMethylation450K BeadChip (Illumina, San Diego, CA)	Genome-wide RR	Significant link found between gestational age, prenatal tobacco exposure, autism polygenic burden, and a −0.14% increase in DNA methylation at specific loci per unit of elevated ASD risk score
Hannon *et al*. [89]	Denmark	Birth weight, gestational age and exposure to maternal smoking	Neonatal dried blood spot	629 ASD cases and 634 TD	Mean gestational age of 39.6 weeks (SD = 1.77 weeks)	ICD-10	Illumina Infinium, HumanMethylation450K BeadChip (Illumina, San Diego, CA)	Genome-wide RR	EWAS linked gestational age and birth weight to 4299 and 18 DMPs, respectively. Maternal smoking associated with 110 DMPs, including *AHRR*, potentially mediating smoking’s impact on birth weight
Potabattula *et al*. [91]	France	N/A	Semen, cord blood, and Peripheral blood	46 ASD 46 TD.	12.7 ± 9.0 years	ADI-R	Pyrosequencing	Candidate gene (*BEGAIN*)	Paternal age negatively correlates with *BEGAIN* promoter methylation in sperm and male offspring’s cord blood, not females. *BEGAIN* hypomethylation, linked to ASD, varies by age, sex, and genetic factors
Gallo *et al*. [92]	Italy	Prenatal exposures	Peripheral blood	42 ASD	4.8 ± 2.0 years	DSM-5 | ADOS-2	MS-HRM	Candidate genes *(BDNF, MECP2, OXTR, HTR1A, RELN, BCL-2 and EN-2)*	High maternal gestational weight gain associated with increased *BDNF* methylation. Lack of maternal folic acid supplementation and low *RELN* methylation associated with higher severity of ASD
Rijlaarsdam *et al*. [93]	Netherlands	Prenatal stress exposure	Cord blood	743 with *OXTR* DNA methylation and autistic traits	N/A	SRS	Illumina Infinium, HumanMethylation450K BeadChip (Illumina, San Diego, CA)	Candidate gene *(OXTR)*	The Generation R Study found that prenatal maternal stress exposure was associated with child autistic traits, but not *OXTR* methylation*. OXTR* methylation levels were positively associated with social problems in G-allele homozygous children, but not A-allele carriers
Stoccoro *et al*. [94]	Italy	Prenatal exposures	Peripheral blood	58 ASD	4.35 ± 1.79 years	ADOS-2	MS-HRM	Candidate genes (*BDNF, MECP2, OXTR, HTR1A, RELN, BCL-2 and EN-2)*	Methylation levels of *MECP2, HTR1A and OXTR* genes were connected to females, and those of *EN2, BCL2and RELN* genes to males. High gestational weight gain, lack of folic acid supplements, advanced maternal age, preterm birth, low birthweight and living in rural context were the best predictors of a high ADOS-2 score
Stoccoro *et al*. [95]	Italy	Prenatal exposures	Placenta and buccal cells	28 pregnant women 28 Children	N/A	Not ASD study but looked at some neurodevelopmental genes	MS-HRM	Candidate gene *(LEP*, *MECP2, IGF2, MTHFR, DNMT3B, OXTR, H19-ICR, HSD11B2, BDNF, CYP1A1, ERα, MGMT*, *RELN, NR3C1 and COMT)*	Suboptimal birth weight, maternal stress, and exposure to air pollutants during pregnancy can induce aberrant methylation levels in genes linked to embryogenesis, potentially affecting fetal development, and providing peripheral biomarkers of environmental exposure
Hranilovic *et al*. [192]	Croatia	N/A	Peripheral blood	90 ASD 66 TD	4–45 years	DSM-IV	Sodium bisulfite using the EpiTect Bisulfite Kit and sanger sequencing	Candidate gene *(HTR2A)*	The study found that autistic individuals with the rs6311 AG genotype showed higher mean methylation levels in the *HTR2A* region, suggesting that epigenetic mechanisms may contribute to *HTR2A* dysregulation in individuals with ASD
Martin *et al*. [193]	France	Valproate	N/A	108 parents asked about intra-uterine exposure to valproic acid	N/A	N/A	N/A	N/A	Among their 187 children they reported 43 (23%) children with malformations and 82 (44%) children with neurodevelopmental disorders (63 problematic behaviors and autism; 41 psycho-motor disorders; 16 language problems; 16 attention deficit; 5 mental retardation). Only 88 (47%) children had neither malformation nor develop mental disorders
Wieting *et al*. [194]	Germany	N/A	Peripheral blood	20 ASD 20 TD	30.45 ± 7.837 years	ICD-10:F84.5 (Asperger Syndrome) | AAA| German version of the WAIS-IV	Nanopore Cas9-targeted sequencing	Candidate gene *(OXTR)*	No group differences in *OXTR* gene sequence except for rs918316 in the HFA group. Differential methylation analysis of 412 CpG sites showed no significant group-dependent differences
Perini *et al*. [195]	Italy	N/A	Peripheral blood	76 ASD 76 TD Siblings	10.3 ± 4.2 years	DSM IV| BPASS| ADI-R| ADOS| KADIS| .	Illumina Infinium, HumanMethylation BeadChip (Illumina, San Diego, CA)	Genome-wide RR	Significant NK cell reduction in ASD siblings suggests immune imbalance. DMRs affecting neurogenesis, synaptic organization, and immune functions in ASD, with a notable DMR near *CLEC11A and SHANK1*
Cuomo *et al*. [196]	Italy	N/A	Fecal samples	8 ASD 8 TD	32.75 ± 4.02 months	DSM-V	Illumina Infinium, HumanMethylation BeadChip (Illumina, San Diego, CA)	Genome-wide RR	Methylome analysis reveals significant DNA methylation changes in ASD, especially in inflammatory and immune pathways, with *IL-6, IL-1B, TLR3, CXCL13, CXCR3, and DGAT1* showing hypomethylation, indicating enhanced inflammatory status
García-Ortiz *et al*. [197]	Spain	N/A	Peripheral blood	50 ASD and 45 TD	43.7 ± 11.2 months	DSM-5 | ADI-R| ADOS-2 | M-CHAT| PDDBI| CARS| SDQ	MS-HRM	Candidate gene *(LINE-1 regions, NCAM1 and NGF* genes)	Decreased *LINE-1* and increased *NCAM1* methylation in ASD; increased *NGF* methylation in ASD with mental regression during the first two years of life compared to TD and to ASD without mental regression
Garrido *et al*. [77]	Spain	N/A	Semen	36 Fathers of ASD and TD children	N/A	N/A	Methylated DNA immunoprecipitation (MeDIP)	Genome-wide RR	The 805 DMR genomic features were characterized, and their associated genes were identified and linked to ASD and other neurobiology-related genes. The potential sperm DMR biomarkers/diagnostic was validated with blinded test sets (*n* = 8–10) of individuals with an approximately 90% accuracy
Elagoz Yuksel *et al*. [198]	Turkey	N/A	Peripheral blood	27 ASD and 39 TD	Between 22 and 94 months	DSM-IV| CARS	MSRE-PCR	Candidate gene (*OXTR)*	Higher frequency of *OXTR* promoter hypomethylation in ASD
Asia									
Yang *et al*. [96]	China	N/A	Peripheral blood	69 ASD 76 TD	2–6 years	DSM-V| ADI-R| PPVT| ABC| CARS| VABS| SRS| Infant-Junior Middle School Student’s Ability of Social Life Scale.	Methylation pyrosequencing	Candidate gene*(ST8SIA2)*	The methylation levels of the Chr. 15: 92 984 625 and Chr. 15: 92 998 561 sites of the *ST8SIA2* gene in ASD children were higher than those of controls. The Chr. 15: 92 984 625 site was positively correlated with the stereotyped behaviors of ASD children
X. Wang *et al*. [97]	China	N/A	Peripheral blood	54 ASD males	4.24 ± 0.98 years	DSM-IV| ADOS| ADI-R|	Bisulfite sequencing	Candidate gene*(ESR2*)	Researchers found little difference in DNA methylation of *ESR2* gene in autistic individuals, but hypermethylation of specific CpG sites was associated with autistic symptoms severity
Z. Hu *et al*. [99]	China	N/A	Peripheral blood	62 ASD 73 TD	N/A	DSM-IV| CARS	SYBR green‐based quantitative methylation‐specific polymerase chain reaction	Candidate gene*(APOE)*	The study found that *APOE* methylation in peripheral blood DNA is significantly higher in pediatric patients with ASD, suggesting that it could serve as a diagnostic biomarker
Z. Hu *et al*. [98]	China	N/A	Peripheral blood	61 ASD and 66 TD	4.02 ± 2.83 years	DSM-V| CARS| ABC	Quantitative methylation‐specific polymerase chain reaction (qMSP)	Candidate gene(*HTR4*)	Decreased *HTR4* methylation in ASD. The difference was significant in males, but not in females. Higher methylation in females ASD compared to males ASD. No differences between females and males TD subjects
Zhao *et al*. [100]	China	N/A	Peripheral blood	42 ASD 26 TD	4.07 ± 2.78 years	DSM-V| CARS| ABC	qMSP	Candidate gene(*TGFB1, BAX, IGFBP3, PRKCB, PSEN2, CCL2*)	*TGFB1* was found to be significantly hypomethylated in children with autism’s peripheral blood samples, positively associated with the Autism Behavior Checklist interaction ability score
L. Wang *et al*. [101]	China	N/A	Semen	4	N/A	N/A	Single cell bisulfite sequencing	Genome-wide	ZPBS significantly lowers global DNA methylation levels than MSS, enriching DMRs in neurogenesis processes. 47.8% of autism candidate genes are associated with DMRs, mainly due to bivalent chromatin structure
Liang *et al*. [102]	China	N/A	Peripheral blood	30 pairs of monozygotic twins	4–12 years	DSM-V| ADOS	Illumina Infinium, HumanMethylation BeadChip (Illumina, San Diego, CA)	Genome-wide RR	The study found significant enrichment in epigenetic disruption of neurotrophin signaling pathway and gene *SH2B*, supporting DNA methylation differences in ASD etiology
Alshamrani *et al*. [103]	Saudi Arabia	Di(2-ethylhexyl) phthalate	Peripheral blood	28 ASD and 24 TD	7.5 ± 2.9 years old	DSM-V| CARS	ELISA assay	Genome-wide RR	Global DNA hypomethylation in ASD subjects
Algothmi *et al*. [104]	Saudi Arabia	N/A	Peripheral blood	19 ASD and 18 siblings	3–12 years	DSM-V|	DNA methylight qPCR	Candidate gene(*ACSF3)*	The study revealed a significant correlation between *ACSF3* gene expression and specificity protein 1 (SP1) in 17 ASD patient samples, with both genes upregulated in 9 samples and downregulated in 8
Kimura *et al*. [105]	Japan	N/A	Peripheral blood	38 ASD 31 TD	28 ± 6.5 years old	DSM-V| ADOS| ASSQ| WAIS	Illumina Infinium, HumanMethylation BeadChip (Illumina, San Diego, CA)	Genome-wide RR	A potential blood biomarker, cg20793532, was identified in ASD patients, annotated to the hypermethylated and down-regulated *PPP2R2C* gene, providing a potential marker for identifying high-functioning ASD
S. Y. Wang *et al*. [106]	Taiwan	Air pollution	N/A	62 919	N/A	ICD-9 They included three diagnosis codes as ASD: autistic disorder (ICD-9 CM code: 299.0), Asperger syndrome (299.8), and PDD-NOS (299.9)	N/A	N/A	Trimester-specific exposure to CO and NO_2_ significantly increased ASD risk, with the highest hazard ratios in the first trimester for both pollutants
Lee *et al*. [107]	South Korea	Air pollutants (PM_2.5_, CO, SO_2_, NO_2_, and O_3_) heavy metals (Pb, Cd, Cr, Cu, Mn, Fe, Ni, and As)	N/A	*n* = 843 134	N/A	ICD-10 (Autism spectrum disorder (“F84.0–F84.9”), excepting Rett’s syndrome (F84.2)	N/A	N/A	Exposure to SO_2_, NO_2_, and Pb during pregnancy can affect the development of neurologic disorders, such as ASD and epilepsy, depending on the timing of exposure. Further research is needed to understand the relationship between these factors and fetal development
Hamadé *et al*. [108]	Lebanon	Prenatal exposures	N/A	86 ASD 172 TD	12.39 ± 5.92 boys, 10.83 ± 3.23 girls	DSM-IV	N/A	N/A	The study found a significant association between autism and factors such as older parents’ age, male sex, unhappy maternal feelings, living close to industry, and previous childhood infection
George *et al*. [109]	India	Prenatal, natal and postnatal exposures	N/A	143 ASD 200 TD	2-6 years	CARS	N/A	N/A	The study found significant high odds ratios for antenatal, natal, and postnatal risk factors for autism, including excess fetal movement, maternal respiratory infection/asthma, maternal vaginal infection, maternal hypothyroidism, and family history of neuro-developmental disorders
Latin America and the Caribbean									
Aspra *et al*. [118]	Mexico	N/A	Buccal cells	27 ASD and 15 TD	5.2 ± 1.9 years	ADI-R| SRS	Illumina Infinium, HumanMethylation BeadChip (Illumina, San Diego, CA)	Genome-wide RR	The hypermethylation of DMR is associated with the *ZFP57, CPXM2*, and *NRIP2* genes. The hypomethylation of DMRs is associated with the *RASGRF2, GSTT1, FAIM*, and *SOX7* genes
Morales-Marín *et al*. [119]	Mexico	N/A	Buccal cells	29 ASD and 7 TD	5.2 ± 1.1 years	ADI-R| SRS	Illumina Infinium, HumanMethylation BeadChip (Illumina, San Diego, CA)	Genome-wide RR	853 CpGs with differential methylation were found in individuals with ASD. They also discovered 64 genes that were included in the SFARI gene database. The *genes ISM1, PTPRG, SLITRK4, CAP2, and CYP26C1* included the five most statistically significant differentially methylated CpGs
Neri de Souza Reis *et al*. [120]	Brazil	Prenatal, natal and postnatal exposures	Peripheral Blood	67 ASD children and mother pairs	4.7 ± 1.3 years	ADI-R| DSM-V| ICD-10 | CARS	Illumina Infinium, HumanMethylation BeadChip (Illumina, San Diego, CA)	Genome-wide RR	The study used principal component analysis to identify vulnerability components in 67 mothers of autistic children, finding higher correlations with psychosocial stress and biological factors. 11 879 differentially methylated probes were found, indicating environmental and genetic influences
Da Silva *et al*. [121]	Brazil	Prenatal, natal and postnatal exposures	N/A	248 ASD and 886 TD	2–15 years.	DSM-V	N/A	N/A	Meconium in amniotic fluid and cesarean section were associated with increased ASD risk. Emergency cesarean delivery further elevated autism odds, as did multiple adverse peripartum events and obstetric complications
Fezer *et al*. [122]	Brazil	Prenatal, natal and postnatal exposures	N/A	75 ASD	33.7 ± 12.2 months	DSM-V	N/A	N/A	ASD children in the study showed higher rates of prematurity, low birth weight, and perinatal asphyxia compared to national and regional averages in Brazil
Lin *et al*. [123]	Brazil	Prenatal, natal and postnatal exposures	N/A	321 ASD and 236 TD	N/A	DSM-V| M-CHAT| ASQ| CARS	N/A	N/A	Gestational infection strongly linked to severe ASD, with correlations also found between epilepsy, GI symptoms, obesity, and lower cholesterol levels in ASD compared to controls
Maia *et al*. [124]	Brazil	Prenatal, natal and postnatal exposures	N/A	253 ASD and 886 TD	2–15 years.	DSM-V| M-CHAT (Portuguese version)	N/A	N/A	Congenital malformation, newborn jaundice, lack of crying at birth, and childhood seizures were associated with ASD, with a stronger link for those with multiple postnatal problems
Christian *et al*. [125]	Jamaica	Prenatal exposures	N/A	298 ASD and 298 TD	61.3 ± 19.5 months	DSM-IV| ADOS|ADI-R| CARS	N/A	N/A	Mothers exposed to fever, illness, physical trauma, and oil-based paints during pregnancy have a higher risk of having a child with ASD
Rahbar *et al*. [126]	Jamaica	Prenatal exposures	Peripheral blood	65 ASD and 65 TD	65 months	DSM-IV| ADOS| ADI-R| CARS	N/A	N/A	No significant link between blood arsenic levels and ASD was found; however, drinking water sources and consumption of avocado, callaloo, broccoli, or pak choi were tied to higher arsenic concentrations
Rahbar *et al*. [127]	Jamaica	Prenatal exposures	Peripheral blood	65 ASD and 65 TD	65 months	DSM-IV| ADOS| ADI-R| CARS	N/A	N/A	No significant link between blood mercury levels and ASD was observed. Seafood consumption and having parents with high school education were associated with increased mercury exposure in Jamaican children
Oceania									
Noble *et al*. [110]	New Zealand	Tobacco smoking	Peripheral blood	64 Cases exposed to smoke 32 Controls	28–30 years	Conduct Problem Score	BSAS	Candidate genes *(AHRR, ASH2L, BDNF(ASD RELATED),CNTNAP2(ASD RELATED),CYPIAI, DUSP6,GFII, GRIN2B (ASD RELATED),MEF2C, PRDM8)*	Substantial differential DNA methylation of CpG sites in *CYP1A1, ASH2L*, and *MEF2C* in those with conduct problems who had been exposed to smoke in utero
Noble *et al*. [114]	New Zealand and United Kingdom	Prenatal cannabis exposure and tobacco exposure and association with neurodevelopment	Cord blood and peripheral blood	98 (CHDS) 2704 (ALSPAC)	0–40 years	N/A	Illumina Infinium, HumanMethylation BeadChip (Illumina, San Diego, CA).	Genome-wide RR	Significant genome-wide DNA methylation differences at ages 0, 7, 15-17, and 27 linked to prenatal cannabis exposure, alone or with tobacco. Genes *LZTS2, NPSR1, NT5E, CRIP2, DOCK8, COQ5, LPAR5* had common differentially methylated CpG sites
Williams *et al*. [115]	Australia	Prenatal, natal and postnatal exposures	N/A	182 ASD and 89 TD	0–14 years	DSM-IV	N/A	N/A	Factors linked to ASD include being male, preterm birth, older maternal age, mother born outside Australia, and multiple births
Africa									
Weckman *et al*. [128]	Malawi	Malaria	N/A	421 Malawian mother–baby dyads	Evaluation at 1218 and/or 24 months	MDAT| MCAB-CDI	N/A	N/A	Antenatal malaria and maternal immune activation between 33 and 37 weeks’ gestation were linked to delayed language development, with significant reductions in MCAB-CDI language scores
Eskenazi *et al*. [131]	South Africa	DDT	N/A	705 children (93.8%of 752 enrolled mothers)	1–2 years	BSID	N/A	N/A	Increases in cis-DCCA, trans-DCCA, and 3-phenoxybenzoic acid levels were linked to lower Social-Emotional scores at 1 year. Cis-DBCA increases affected Language and Expressive Communication scores, with sex differences in motor function at 2 years
Mohamed *et al*. [132]	Egypt	Mercury, lead and aluminum levels	Hair	100 ASD and 100 TD	6.24 ± 2.4 years	DSM-5 | CARS	N/A	N/A	Increased autism risk was linked to seafood consumption, immunoglobulin D, dental amalgam, painting, old house age, and smoking, older maternal age, and shorter breastfeeding. Autistic children had lower IQs and higher hair levels of mercury, lead, and aluminum
Garrison *et al*. [129]	Benin	Malaria	N/A	493 pregnant women	1 year and 6 years	MSEL	N/A	N/A	A study of 493 pregnant women found that 40% were infected with malaria, with 31% having placental malaria. Impaired gross motor scores were associated with MiP, placental malaria, and high parasite density
Bam *et al*. [133]	South Africa	N/A	Buccal cells	93 ASD 52 TD	6–12 years	ADOS-2	tNGBS	Candidate genes *(PGC-1α, STOML2, FIS1, MFN2, OPA1*, and *GABPA)*	*PGC-1α*, a transcriptional regulator, was significantly hypermethylated at eight CpG sites. Mitochondrial DNA copy number was elevated in ASD, correlated with methylation at the *PGC-1α* promoter. This suggests differential methylation in ASD
El-Baz *et al*. [199]	Egypt	Copper	Peripheral blood	20 ASD 20 TD	6.15 ± 3.133 years	ICD-10 | CARS	N/A	N/A	Patients with ASD showed more stereotypic movements, absent eye contact, delayed motor/speech development, and lower IQ than controls, with significantly higher levels of copper and ceruloplasmin
Meguid *et al*. [200]	Egypt	Prenatal, natal and postnatal exposures	N/A	530 ASD	1–15 years	DSM-V| ADI-R| CARS	N/A	N/A	Caesarean section was the most common risk factor (57.4%), followed by jaundice (30.1%)
Sangare *et al*. [51]	Mali	N/A	N/A	105 ASD	3–14 years	DSM-V| ICD-10	N/A	N/A	Autistic children were born to first degree consanguineous marriage and a multipara woman (>7 births) with a family history of psychiatric disorder on the paternal side two times as frequently as compared to children with epilepsy
Omotosho *et al*. [148]	Nigeria	Heavy metals	Peripheral blood	25 ASD 25 TD	5.25 ± 0.37 years	DSM-IV	N/A	N/A	Pb (lead) concentration was significantly increased while Mg, Zn, and Cu levels were reduced significantly in ASD compared to TD
Stathopoulos *et al*. [201]	South Africa	N/A	Buccal cells	93 ASD 52 TD	6–12 years	ADOS-2	Illumina Infinium, HumanMethylation BeadChip (Illumina, San Diego, CA).	Genome-wide RR	Differentially methylated CpG sites in ASD mapped to 898 genes, affecting mitochondrial metabolism and protein ubiquitination pathways. PCCB and *PCDHA12* showed significant methylation differences, with hypomethylation in ASD
Feil *et al*. [202]	South Africa	Prenatal exposures	Cord blood	142 mother-child pairs	BSID score measured at 2 years of age	BSID	Illumina Infinium, HumanMethylation BeadChip (Illumina, San Diego, CA).	Genome-wide RR	29 CpG sites and 4 genes *(GOPC, RP11-74K11.1, DYRK1A, RNMT)* were identified as significant mediators of the association between PM10 and cognitive neurodevelopment
Mankoski *et al*. [203]	Tanzania	Malaria	N/A	14 ASD	12.3 years	ADI-R(Kiswahili)	N/A	N/A	Three children with normal development experienced autism upon recovery from severe malaria, accompanied by high fever, convulsions, and loss of consciousness. In four cases, the relationship between autism and infection was close, possibly suspicious due to malaria’s prevalence in Tanzania
Slama *et al*. [204]	Tunisia	Prenatal, natal and postnatal exposures	Peripheral blood	51 ASD 40 TD	7.30 ± 3.2 years	DSM-V| CARS| ADOS-2 | ADI-R	N/A	N/A	Breastfeeding for less than 6 months, older paternal age at childbirth, low blood cholesterol, and low erythrocyte magnesium levels were linked to increased ASD risk compared to TD
Hadjkacem *et al*. [205]	Tunisia	Prenatal, natal and postnatal exposures	N/A	50 ASD 51 TD Siblings	3–7 years	DSM-5 | CARS	N/A	N/A	Higher prevalence of prenatal, perinatal, and postnatal factors in ASD children included fetal distress, long delivery, prematurity, and respiratory infections, with no parental age correlation. Risk factors also included male gender and prenatal UTI

Abbreviations: AAA: Adult Asperger Assessment; ABC: Autism Behavior Checklist; ADHD: Attention-deficit/hyperactivity disorder; ADI-R: Autism Diagnostic Interview-Revised; AI: Artificial Intelligence; ALSPAC: Avon Longitudinal Study of Parents and Children As: Arsenic; ASQ: Ages and Stages Questionnaires; Autism Spectrum Screening Questionnaire; AUC: Area Under Curve; BPASS: Broader Phenotype Autism Symptom Scale; BSAS: bisulfite-based amplicon sequencing; BSID: Bayley Scales of Infant Development; CARS: Childhood Autism Rating Scale; Cd: Cadmium; CHARM: Comprehensive High-throughput Arrays for Relative Methylation; CHDS: Christchurch Health and Development Study; CO: Carbon Monoxide; Cr: Chromium; Cu: Copper; DDE: Dichlorodiphenyldichloroethylene; DDT: Dichlorodiphenyltrichloroethane; DSM-IV/DSM-V: Diagnostic and Statistical Manual of Mental Disorders, Fourth Edition/Fifth Edition.; ELISA: enzyme-linked immunosorbent assay; Fe: Iron; FXS: Fragile X syndrome; GI: Gastrointestinal; ICD-9/ICD-10: International Classification of Diseases, Ninth Revision/ Tenth Revision; IQ: Intelligence Quotient; KADIS: Krug Asperger Disorder Index screening; M-CHAT: Modified Checklist for Autism in Toddlers; MCAB-CDI: MacArthur–Bates Communicative Development Inventories; MDAT: Malawi Developmental Assessment Tool; meQTLs: methylation Quantitative Trait Loci; Mn: Manganese; MS-HRM: Methylation-Sensitive High-Resolution Melting; MSEL: Mullen scales of early learning; Manually Selected Spermatozoa; N/A: Not Applicable; Ni: Nickel; NO_2_: Nitrogen Dioxide; O_3_: Ozone; Pb: Lead; PCB: Polychlorinated Biphenyls; PBDEs: Polybrominated Diphenyl Ethers PDDBI: Pervasive Developmental Disorders Behavior Inventory; PM_2.5_: Particulate Matter 2.5; PMD: Partially methylated domains; PPVT: Peabody Picture Vocabulary Test; qMSP, quantitative methylation-specific PCR; RR: Reduced Representation; RRBS: reduced-representation bisulfite sequencing; SDQ: Strengths and Difficulties Questionnaire; SFARI: Simons Foundation Autism Research Initiative; SO_2_: Sulfur dioxide; TD: Typical Development; tNGBS: targeted Next-Generation Bisulfite Sequencing; THC: Tetrahydrocannabinol, UTI: Urinary Tract Infection; VABS: Vineland Adaptative Behavior Scale; WAIS-IV: Wechsler Adult Intelligence Scale—IV.

**Table 2.** Key findings on global ASD prevalence and additional relevant studies

Study	Country	Region	Sample size/denominator	ASD total cases	Age (years)	ASD/neurodevelopment diagnostic tools	Sex ratio (M:F)	Prevalence estimate per 10 000	Data collection year
North America									
Autism and Developmental Disabilities Monitoring Network Surveillance Year 2000 Principal Investigators [25]	USA	Arizona, Georgia, Maryland, South Carolina, West Virginia	187 761	1252	8	DSM-IV| ICD-9	4.3:1	67	2000
Autism and Developmental Disabilities Monitoring Network Surveillance Year 2002 Principal Investigators [149]	USA	Arizona, Arkansas, Colorado, Georgia, Maryland, Missouri, New Jersey, North Carolina, Pennsylvania, South Carolina, Utah, West Virginia, Wisconsin	407 578	2685	8	DSM-IV| ICD-9	5.5:1	66	2002
Autism and Developmental Disabilities Monitoring Network Surveillance Year 2006 Principal Investigators & Centers for Disease Control and Prevention (CDC) [150]	USA	Alabama, Arizona, Colorado, Florida, Georgia, Maryland, Missouri, North Carolina, Pennsylvania, South Carolina, and Wisconsin	307 790	2757	8	DSM-IV| ICD-9	4.9:1	90	2006
Ouellette-Kuntz *et al*. [26]	Canada	Manitoba, Southeastern Ontario, Prince Edward Island, and Newfoundland and Labrador.	89 786	1173	2–14	Clinical	4.8:1	130.6	2003–10
Autism and Developmental Disabilities Monitoring Network Surveillance Year 2008 Principal Investigators & Centers for Disease Control and Prevention [151]	USA	Alabama, Arizona, Arkansas, Colorado, Florida, Maryland, Missouri, New Jersey, North Carolina, Pennsylvania, South Carolina, Utah, West Virginia, and Wisconsin	337 093	8	3820	DSM-IV| ICD-9	4.6:1	113	2008
Developmental Disabilities Monitoring Network Surveillance Year 2010 Principal Investigators & Centers for Disease Control and Prevention (CDC) [152]	USA	Alabama, Arizona, Arkansas, Colorado, Georgia, Maryland, Missouri, New Jersey, North Carolina, Utah, and Wisconsin	363 749	5338	8	DSM-IV| ICD-9	4.5:1	147	2010
Christensen *et al*. [153]	USA	Arkansas, Arizona, Colorado, Georgia, Maryland, Missouri, New Jersey, North Carolina, South Carolina, Utah, and Wisconsin	346 978	5063	8	DSM-IV| ICD-9	4.5:1	146	2012
Ofner *et al*. [27]	Canada	Newfoundland and Labrador, Nova Scotia, Prince Edward Island, New Brunswick, Quebec, British Columbia, and the Yukon	1 916 588	29 099	5–17	DSM-V	4:1	152	2015
Baio *et al*. [154]	USA	Arizona, Arkansas, Colorado, Georgia, Maryland, Minnesota, Missouri, New Jersey, North Carolina, Tennessee, and Wisconsin	325 483	5473	8	DSM-IV| DSM-V| ICD-9	4:1	168	2014
Diallo *et al*. [155]	Canada	Quebec	16 940	1–17	207	ICD-9 | ICD-10	3.8	122	2014–15
Maenner *et al*. [156]	USA	Arizona, Arkansas, Colorado, Georgia, Maryland, Minnesota, Missouri, New Jersey, North Carolina, Tennessee, and Wisconsin	275 419	5108	8	DSM-IV| DSM-V| ICD-9 | ICD-10	4.3:1	185	2016
Maenner *et al*. [157]	USA	Arizona, Arkansas, California, Georgia, Maryland, Minnesota, Missouri, New Jersey, Tennessee, Utah, and Wisconsin	220 281	5058	8	DSM-V| ICD-9 | ICD-10	4.2:1	230	2018
Maenner *et al*. [3]	USA	Arizona, Arkansas, California, Georgia, Maryland, Minnesota, Missouri, New Jersey, Tennessee, Utah, and Wisconsin	226 339	6245	8	DSM-V| ICD-9 | ICD-10	3.8:1	276	2020
Europe									
Morales-Hidalgo *et al*. [29]	Spain	Tarragona	2755	37	3–5	Parent and/or teacher report| CAST| ADI-R| ADOS-2	4.3:1	155	
Morales-Hidalgo *et al*. [29]	Spain	Tarragona	2827	34	10–12	Parent and/or teacher report| CAST| ADI-R| ADOS-2	4.4:1	100	
Ellefsen *et al*. [158]	Denmark	Faroe Islands	7689	41	8–17	ICD-10 | Gillberg criteria	6:1	56	2002
Isaksen *et al*. [159]	Norway	Oppland and Hedmark	31 015	158	6–12	ICD-10 | ADOS| ADI	4.27	51	2002–8
Nygren *et al*. [160]	Sweden	Göteborg	5007	40	2	DSM-IV-TR	4	80	2010
Kočovská *et al*. [161]	Denmark	Faroe Islands	7128	67	15–24	ICD-10 | DSM-IV| Gillberg criteria	2.7	94	2009
Saemundsen *et al*. [162]	Iceland	Nationwide	22 229	267	6	ICD-10 | ADOS| ADI	2.8	120.1	2005–9
Idring *et al*. [163]	Sweden	Stockholm	735 096	11 330	0–27	DSM-IV|ICD-9 | ICD-10	2.3	154	2011
van Bakel *et al*. [164]	France	Isère, Savoy, Upper-Savoy, and Haute-Garonne counties	307 751	1123	7	ICD-10	4.1	36.5	1997–3
Bachmann *et al*. [165]	Germany	Nationwide	6 900 000	14 749	0–24	Registry-based| ICD-10	1.7:1	22	2006
Bachmann *et al*. [165]	Germany	Nationwide	6 400 000	21 186	0–24	Registry-based| ICD-10	2.8:1	38	2012
Skonieczna‐Żydecka *et al*. [166]	Poland	West Pomerania and Pomerania	708 029	2514	0–16	ICD-10	4.3	35	2010–14
Delobel-Ayoub *et al*. [31]	Denmark	Nationwide	195 293	2414	7–9	registry-reported| DSM-IV	3.9	124	2015
Delobel-Ayoub *et al*. [31]	France	Isère, Savoie, and Haute-Savoie	32 342	154	8	registry-reported| ICD-10	4	48	2015
Delobel-Ayoub *et al*. [31]	France	Haute-Garonne county	15 836	115	8	registry-reported| ICD-10	5.4	73	2015
Delobel-Ayoub *et al*. [31]	Finland	Nationwide	177 193	1347	7–9	registry-reported| DSM-IV	3.3	76	2015
Delobel-Ayoub *et al*. [31]	Iceland	Nationwide	13 551	363	7–9	registry-reported| ICD-10	4.4	268	2015
Narzisi *et al*. [32]	Italy	Pisa	10 138	81	7–9	TN| SCQ| ADOS	5.25:1	115	2014–16
Thomaidis *et al*. [167]	Greece	Nationwide	182 879	2108	3–10	ICD-10 | DSM-V	4.14	115	2019
Fuentes *et al*. [33]	Spain	Gipuzkoa	14 734	65	7–9	ADOS| ADI-R| DSM-IV| DSM-5	6.2:1	59	2017–18
Roman-Urrestarazu *et al*. [168]	England	Nationwide	7 047 238	119 821	5–19	Registry-based	4.32:1	176	2017
Rasga *et al*. [34]	Portugal	Central Region	13 690	55	7–9	ADOS| ADI-R| DSM-V	2.9:1	50	2016–17
O’Nions *et al*. [169]	England	Nationwide	602 433	4704	0–70	Registry-based	3.4:1	78	2018
O’Nions *et al*. [169]	England	Nationwide	5 586 100	12 098	0–70	Registry-based	3.8:1	22	2018
Dinstein *et al*. [170]	Israel	Nationwide	3 014 500	14 914	1–17	Registry-based| DSM_IV| DSM-V		49	2017
Dinstein *et al*. [170]	Israel	Nationwide	3 071 500	18 189	1–17	Registry-based| DSM_IV| DSM-V		59	2018
Dinstein *et al*. [170]	Israel	Nationwide	3 125 700	22 697	1–17	Registry-based| DSM_IV| DSM-V		73	2019
Dinstein *et al*. [170]	Israel	Nationwide	3 239 300	27 807	1–17	Registry-based| DSM_IV| DSM-V		86	2020
Dinstein *et al*. [170]	Israel	Nationwide	3 360 200	32 222	1–17	Registry-based| DSM_IV| DSM-V		96	2021
Asia									
Lai *et al*. [171]	Taiwan	Nationwide	4 664 310	3995	3–17	Registry-reported	5.75:1	8.5	2004
Lai *et al*. [171]	Taiwan	Nationwide	4 601 833	4684	3–17	Registry-reported	5.64:1	10.1	2005
Lai *et al*. [171]	Taiwan	Nationwide	4 487 827	5345	3–17	Registry-reported	5.85:1	11.9	2006
Lai *et al*. [171]	Taiwan	Nationwide	4 395 283	6119	3–17	Registry-reported	5.97:1	13.9	2007
Lai *et al*. [171]	Taiwan	Nationwide	4 268 630	6771	3–17	Registry-reported	6:1	15.9	2008
Lai *et al*. [171]	Taiwan	Nationwide	4 158 210	7479	3–17	Registry-reported	6.06:1	18	2009
Lai *et al*. [171]	Taiwan	Nationwide	4 044 433	8072	3–17	Registry-reported	6.06:1	19.9	2010
Zhou *et al*. [36]	China	Nationwide	125 806	363	6–12	ADOS| ADI-R| DSM-V	4.3:1	29	2014–16
Sasayama *et al*. [172]	Japan	Nationwide	6262, 731	172 276	1–10	ICD-10	2:1	270	2009–16
Chaaya *et al*. [142]	Lebanon	Beirut, Mount-Lebanon	998	263	1.3–4	M-CHAT + short questionnaire	1.05	153	2014
Raina *et al*. [173]	India	Urban, rural, and tribal	28 070	43	1–10	ISAA	1.15	15	
Jin *et al*. [174]	China	Shanghai	74 252	203	3–12	SCQ| DSM-V	3.6	7.58	2014
Akhter *et al*. [175]	Bangladesh	Sirajganj district	5286	4	1.5–3	MCHAT| ADOS| DSM-IV	3	7.5	2016
Heys *et al*. [176]	Nepal	Makwanpur	4098	14	9–13	AQ-10	1.4	30	2014–15
Hong *et al*. [177]	South Korea	Nationwide	51 529 338	5653	0–89	ICD-10	5.4:1	1.09	2008–15
Hoang *et al*. [178]	Vietnam	Hanoi Capital + 2 northern provinces	17 277	130	1.5–2.5	M-CHAT| DSM-IV	4.65	75.2	2017
Alshaban *et al*. [179]	Qatar	Nationwide	133 781	1099	6–11	DSM-V	4.26	114	2015–18
Al-Mamari *et al*. [180]	Oman	Muscat	837 655	1705	0–14	DSM-V	3.4	20.35	2011–18
Sabbagh *et al*. [181]	Saudi Arabia	Makkah and Jeddah	347 036	1023	8.95 ± 2.39	DSM-V	3.5:1	28.1	2020
Oceania									
May *et al*. [182]	Australia	Nationwide	3300	145	12–13	Parent| teacher report	3:1	436	2016
May *et al*. [182]	Australia	Nationwide	3913	98	12–13	Parent| teacher report	3:1	260	2016
Drysdale & van der Meer [183]	New Zealand	Hutt Valley Region	228	33	0–19	N/A	4:1	14.8	2012–16
Bowden *et al*. [38]	New Zealand	Nationwide	165 292	9555	0–24	DSM-IV| ICD-10	3.6:1	57.4	2015–16
Latin America									
Montiel-Nava & Peña [42]	Venezuela	Maracaibo	254 905	430	3–9	DSM-IV-TR| ADOS| CARS	3.25:1	17	2005–6
Paula *et al*. [39]	Brazil	Atibaia	1470	4	7–12	DSM-IV| ADI-R	4:0	27.2	N/A
Dekkers *et al*. [43]	Ecuador	Quito	51 453	57	5–15	DSM-III| DSM-IV	4.7	11.1	N/A
Fombonne *et al*. [44]	Mexico	Guanajuato	12 116	36	8	SRS| ADI-R| ADOS-G	4:1	87	2011–12
García-Zambrano *et al*. [41]	Colombia	Nationwide	9 981 158	18 695	0–14	Registry- Reported	N/A	18.7	2009–19
Roman-Urrestarazu *et al*. [40]	Chile	Nationwide	3 056 306	14 549	6–18	Registry- Reported	6.35:1	46	2014–21
Africa									
Seif Eldin *et al*. [46]	Egypt	N/A	122	41		M-CHAT	4:1	3360	2006–7
Seif Eldin *et al*. [46]	Tunisia	N/A	122	14		M-CHAT	4:1	1150	2006–7
Zeglam & Maouna [47]	Libya	Tripoli	38 508	128	0–16	DSM-IV	4:1	3	2005–9
Kakooza-Mwesige *et al*. [48]	Uganda	Kampala and Wakiso	1169	2–9	8	23Q| DSM-IV-TR	1.2	120	2010–11
Chinawa *et al*. [49]	Nigeria	Enugu and Ebonyi	721	21	3–18	parent report	1	290	2014
Sangare *et al*. [51]	Mali	Bamako	2343	105	3–14	DSM-V| ICD-10	1.5:1	450	2014
Pillay *et al*. [50]	South Africa	Western Cape	1 154 353	940	3–23	Registry-Based	5.5:1	8	2016

Abbreviations: 23-Q: 23-question screener; ADOS-G: Autism Diagnostic Observation Schedule—; ADI-R: Autism Diagnostic Interview-Revised; AQ: Autism-Spectrum Quotient; CARS; Childhood Autism Rating Scale; CAST: Childhood Autism Spectrum Test; DSM-III/DSM-IV/DSM-IV-TR/DSM-V: Diagnostic and Statistical Manual of Mental Disorders, Third Edition/Fourth Edition/Fourth Edition Text Revision/Fifth Edition.; ICD-9/ICD-10: International Classification of Diseases, Ninth Revision/ Tenth Revision; ISAA: Indian Scale for Assessment of Autism; M-CHAT: Modified Checklist for Autism in Toddlers; N/A: Not Applicable; SCQ: Social Communication Questionnaire; TN: Teacher Nomination.

## Methodology

A comprehensive search was conducted using electronic databases, including PubMed, Scopus, and Google Scholar, to gather relevant literature for this review. The search terms included “Autism Spectrum Disorder,” “Autistic Disorder,” “ASD,” “Asperger’s Syndrome,” “Pervasive Developmental Disorder,” “Child Disintegrative Disorder,” “neurodevelopmental disorder,” “DNA methylation,” “environmental factors,” “prevalence,” and “epigenetics.” Studies were selected based on their relevance to the intersections of environmental factors, DNA methylation, and ASD. Articles were included if they were peer-reviewed, written in English, and provided data on human subjects. We focused on including studies from all continents to ensure a global perspective. Additionally, references from selected articles were reviewed to identify further relevant studies. The final selection included studies that provided insights into the environmental and epigenetic aspects of ASD, with a specific focus on DNA methylation patterns as potential biomarkers. Our aim in this section was to include studies encompassing all three components: ASD, environmental factors, and DNA methylation, but exceptions were made to include those with two components if they were from regions outside of North America or Europe.

Lastly, to highlight the heterogeneity of ASD diagnosis, we also examined prevalence estimates from different geographic regions worldwide. We will initially discuss the prevalence, followed by the environmental factors from the different regions.

### Prevalence of ASD: a comparative analysis across continents

According to the Centers for Disease Control and Prevention, the prevalence of ASD in children in the USA was 1 in 150 in 2000 to 1 in 36 in 2020, with higher rates in males [3, 25]. In Canada, ASD prevalence was 1 in 70 between 2003 and 2010 [26]. However, the National Autism Spectrum Disorder Surveillance System reported that 1 in 66 children were affected in 2015, with males affected more [27].

The average prevalence of ASD in Europe is currently around 1% [28]. However, there is variability in the prevalence due to different study groups utilizing different diagnostic tools, age groups, sample sizes, and an underestimation of female prevalence. Prevalence estimates from the ASD in the European Union project ranged from 0.48% to 2.68%, while Spain ranged from 1.00% to 1.55% [29–34]. Countries in Europe benefit from well-established national ASD registries.

According to a meta-analysis by Qiu *et al*., the prevalence of ASD in Asia was 0.36% [35]. Compared to West Asia (0.35%) and South Asia (0.31%), the prevalence of ASD in East Asia was the highest (0.51%). In China, between 2014 and 2016, the prevalence was 0.29% [36].

The Australian Bureau of Statistics reported that the number of ASD cases increased to 290 900 in 2022 from 205 200 in 2018, with males being affected more. This was a 41.8% increase [37]. In New Zealand, a prevalence estimate of 1 in 102 was found in 2020 [38].

Latin America and Africa face significant challenges due to a lack of extensive research on ASD. Prevalence data are limited and often derived from localized studies, as there are no comprehensive national ASD registries in these regions. This results in fragmented and regional estimates rather than a complete picture of the disorder’s impact on a national scale. In Latin America, the prevalence ranged from 0.27% to 0.87% prevalence [39–44]. In Africa, the prevalence ranged from 0.08% to 33.6% [45–50]. High consanguinity rates in some regions may increase genetic risk, but cultural stigma and limited healthcare access hinder diagnostic accuracy [4, 51].

The prevalence of ASD varies widely across different geographic regions and changes over time, reflecting the influence of diverse environmental, genetic, and social factors. This variability underscores the need for further investigation into how DNA methylation studies may help to provide insights into the molecular mechanisms of diverse genetic and environmental factors contributing to ASD prevalence across time and place.

### Environmental factors associated with ASD and DNA methylation by continent

#### North America

North American research on the intersection between environmental factors and ASD is concentrated in the USA and Canada. The brain is the ideal tissue for research on ASD since it is a neurodevelopmental disorder. Numerous studies identified DNA methylation changes in brain tissue from ASD patients [52–62]. However, these studies were inherently limited in sample size and because of the difficulties in establishing connections with environmental factors. For this reason, studies performed on perinatal and peripheral tissues as surrogates for the brain are appropriate as these tissues are more accessible, and connections with environmental factors and DNA methylation can be analyzed. In the USA, two prospective ASD enriched-risk studies have been important. Markers of Autism Risk in Babies-Learning Early Signs (MARBLES) is a longitudinal birth cohort at an enriched risk for ASD because of recruitment from mothers with at least one child diagnosed with ASD [63]. The Early Autism Longitudinal Investigation (EARLI) is a similar cohort study that tracks pregnancies at high risk for ASD. Both studies seek to identify early environmental and genetic risk factors associated with ASD.

The placenta is an appropriate tissue for studying the impact of environmental variables on neurodevelopment because of its crucial function in regulating maternal–fetal interactions and its role as a biological repository of prenatal environmental exposures [64–70]. An early MARBLES placental DNA methylation study found that self-reported exposure to professionally applied pesticides during pregnancy was associated with changes in placental DNA methylation in children with ASD compared to those with typical development (TD) [68]. Specifically, it increased methylation in placental partially methylated domains (PMDs), suggesting a global impact on placental DNA methylation.

Cord blood is also an accessible and valuable perinatal tissue because it directly represents the infant’s prenatal environment and can offer insights into early developmental changes influenced by environmental factors. Another study examined the link between air pollution and placenta and cord blood in mothers of infants with ASD [69]. The study revealed four differentially methylated regions (DMRs) in cord blood at the genes *RNF39, CYP2E1*, and *PM20DI*, and five DMRs in the placenta at the genes *ZNF442, PTPRH, SLC25A44, F11R*, and *STK38*. Additionally, they discovered female-specific changes in cord blood methylation at the *CYP2E1* gene that were explicitly related to NO_2_ exposure. Furthermore, they found male-specific changes in methylation at the *RNF39* gene locus in response to O_3_ exposure in cord blood, while females only showed female-specific modifications at the *PM20D1* gene locus. They also discovered a substantial shift in methylation at the *F11R* gene locus in the placenta of male offspring alone, which was linked to NO_2_ exposure. Previous studies have shown that some of these genes have a role in immunological and inflammatory processes in biology [71, 72], and *CYP2E1* was also identified as differentially methylated in ASD placenta from a different cohort [65].

Aung *et al*. investigated potential associations between maternal blood metal concentration and whole blood methylation using a subsample from this cohort [73]. Significant hypermethylation was detected at 11 DNA methylation loci close to the genes *CYP24A1, ASCL2, FAT1, SNX31, NKX6-2, LRC4C, BMP7, HOXC11, PCDH7, ZSCAN18*, and *VIPR2*, which were all associated with lead exposure. These genes were enriched for biological pathways such as cell adhesion, nervous system development, and calcium ion binding. Four DNA methylation loci were also discovered to be associated with manganese hypermethylation and were enriched for cellular metabolic pathways. These pathways play critical roles in neurodevelopment and functioning, which are often disrupted in ASD. Cell adhesion is essential for forming and maintaining neural connections, while nervous system development and cellular metabolism are required for neurons’ proper growth and maturation. Calcium ion binding is crucial for neurotransmission and intracellular signaling. Dysregulation in these pathways can lead to impaired neural connectivity and communication, which are hallmark features of ASD.

Persistent organic pollutants (POPs) like polychlorinated biphenyl (PCBs) and polybrominated diphenyl ethers (PBDEs) are suspected contributors to neurodevelopmental disorders because they can disrupt endocrine and neurological functions, leading to developmental delays and cognitive impairments. Their ability to accumulate in the environment and human tissues poses a significant risk to fetal brain development [56, 70, 74]. A study of MARBLES placental methylation used correlated methylation modules and found two modules linked to maternal PCB levels and child neurodevelopment [70, 74]. These modules matched to genes *AUTS2* and *CSMD1*, previously linked to ASD [75, 76] and PCB exposure [74]. According to their results, the mother’s age, the year the sample was collected, her pre-pregnancy BMI, and her levels of polyunsaturated fatty acids were the best indicators of PCB levels. Mitchell *et al*. investigated the levels of seven polybrominated diphenyl ethers (PBDEs) and eight PCBs [56]. The researchers used postmortem brain tissues from a variety of subjects, including 43 neurotypical controls, 32 individuals with known genetic causes of neurodevelopmental disorders (such as Down syndrome, Rett syndrome, Prader-Willi, Angelman, and 15q11-q13 duplication syndromes), and 32 individuals with idiopathic autism. Compared to neurotypical controls, those with 15q11-q13 duplication syndrome had much higher levels of PCB 95, whereas those with idiopathic ASD did not.

Sperm tissue has also been used to study the paternal influence of genetics and environment on ASD prevalence [77–83]. Paternal autistic traits and the sperm epigenome are connected to ASD because epigenetic modifications in sperm can influence gene expression in offspring, potentially contributing to ASD risk. The sperm epigenome is crucial as it carries heritable epigenetic marks that can affect children’s early developmental processes and neurodevelopmental outcomes. An investigation explored the potential link between autistic traits in children as young as 36 months from the EARLI cohort, paternal autistic characteristics, and the sperm epigenome [80]. The study utilized the Social Responsiveness Scale (SRS), a 65-item questionnaire that measures social communication deficits and autistic traits. It identified 14 paternal and 94 child SRS-associated DMRs. Many child-associated DMRs were connected to genes essential for ASD and neurological development. Additionally, five DMRs overlapped between children and their fathers, involving genes *WWOX, SALL3, AJAP1, TGM3*, and *IRX4*, which are significant in ASD research.

Schrott *et al*. performed several investigations to understand how cannabis affects DNA methylation. One study used a candidate gene approach based on sperm *DLGAP2* DNA methylation previously associated with ASD [54, 82], confirming that sperm from cannabis users showed differential methylated CpG sites in *DLGAP2* compared to controls [83]. Interestingly, *DLGAP2* was associated with changes in DNA methylation in newborns due to maternal smoking in pregnancy in another study [84]. Bisulfite pyrosequencing on nine clustered CpG sites revealed hypomethylation linked to cannabis use. Cannabis was also associated with changes in DNA methylation at autism candidate genes and maternally imprinted genes in spermatogenic stem cells [81]. In spermatogenic stem cells, cannabis exposure significantly impacted the methylation of 2 out of 10 ASD candidate genes, *NR4A2* and *HCN1*. In addition, spermatid-like cells showed considerably differential methylation of *PEG3*, and spermatogenic stem cells showed significantly altered methylation of maternally imprinted genes *SGCE* and *GRB10*.

Researchers in Canada looked for evidence of DMRs in ASD patients compared to controls using candidate gene approaches. Environmental influences were not examined. A study of neurodevelopmental disorders and DNA methylation of the oxytocin receptor was the subject of one research study [85]. The group they studied consisted of individuals with ASD, attention-deficit/hyperactivity disorder (ADHD), and obsessive-compulsive disorder (OCD). Individuals with ASD, ADHD, or OCD showed differential DNA methylation at specific locations in the first intron of *OXTR* in their blood or saliva.

Additionally, compared to those whose DNA methylation patterns fell within the normal ranges for each respective neurodevelopmental disorder group, people with ASD or ADHD showed the most extreme DNA methylation values at specific sites, which were also associated with higher scores on the Child Behavior Checklist (CBCL) social problems subscale (ADHD) or lower IQs (ASD). Their findings demonstrated a complicated, measurable link between neurodevelopmental disorders and *OXTR* DNA methylation. Another study by Siu *et al*. aimed to identify DNA methylation signatures for ASD subgroup molecular classification [86]. They found that 16p11.2 and *CHD8* subgroups had unique DNA methylation signatures that distinguished them from each other and idiopathic ASD and controls, providing a more precise classification and potential for developing diagnostic biomarkers for the subgroups.

This comprehensive overview of studies from the USA and Canada highlights the complex relationship between environmental factors, DNA methylation, and ASD, revealing the potential use of peripheral tissues like the placenta and sperm to provide insights into the early developmental basis of neurodevelopmental disorders.

#### Europe

Using organized cohorts, several studies looked at environmental risk factors that are thought to be linked to ASD. These included endocrine-disrupting compounds (EDCs), POPs, and maternal smoking. A study in the Faroe Islands, Denmark, performed sperm methylome analysis on 52 samples and assessed the effects of exposure to 1,1-dichloro-2,2-bis(p-chlorophenyl) ethylene (DDE), a banned insecticide [79]. This is particularly interesting because the population in these regions is known to consume whale meat with high levels of POPs. Whole-genome bisulfite sequencing (WGBS) revealed that genes *CSMD1, NRXN2*, and *RBFOX1* exhibit hypomethylation across individual samples [79]. Genes *CSMD1* and *NRXN2* are highly expressed in the brain and are associated with neuro-vertebrate development, which is linked with developmental delay phenotypes in ASD by the SFARI database [76, 87].

Furthermore, *SNORD115-30* and *SNORD115-37*, which are in an imprinted region, exhibit hypermethylation and were consistently observed to be hypermethylated from a previous study on paternal sperm samples within an enriched risk for ASD cohort [78, 79]. In another study, *PTPRN2* showed hypomethylation in cord blood, which correlated with the levels of exposure to DDE [88]; however, in the study by Maggio *et al*., *PTPRN2* transcript levels showed no correlation with levels of DDE, and samples showed both hyper and hypomethylated DDE DMRs, which signifies a potential role with DDE and epigenetic alterations linked to ASD.

Maternal smoking is another significant environmental risk factor explored in European studies. Two studies from Denmark performed an epigenome-wide association study (EWAS) focusing on gestational age, birth weight, and maternal smoking, identified altered differentially methylated positions (DMPs) in ASD children compared to non-ASD children [89, 90]. Specifically, they identified 4299 DMPs associated with gestational age, 18 DMPs with birth weight, and 110 DMPs with maternal smoking [90]. Genes such as *AHRR, GFI1*, and *EXOC2* methylation sites were associated with maternal smoking and birth weight [89, 90]. These studies benefit from extensive and unbiased sample sizes through Denmark’s comprehensive neonatal screening program. However, it is essential to note that the methylation studies used a small subset of participants, so they may not necessarily be nationally representative or have increased power over other studies.

A candidate gene approach study examined paternal age’s impact on the *BEGAIN* gene’s methylation status in sperm samples [91]. They found that the ASD population showed hypomethylation of *BEGAIN* compared to neurotypical controls. They also observed paternal age-associated *BEGAIN* methylation in male fetal cord blood but not in female fetal cord blood. This candidate gene is intriguing because *BEGAIN* is one of the few known autosomal genes with sex specificity that contributes to dimorphic traits and disease susceptibility in ASD. While functional and mechanical changes associated with the *BEGAIN* gene are unknown, it at least represents the elevated risk for ASD in children from older fathers.

The methylation status of important candidate genes, including *MECP2, OXTR, BDNF, RELN, BCL2, EN2*, and *HTR1A*, was examined in young females with respect to various risk factors, such as maternal age, pre-pregnancy BMI, gestational age, and delivery methods [92, 93]. They found that high maternal gestational weight gain was significantly associated with hypermethylation of *BDNF*, and maternal folic acid supplementation correlated with hypomethylation in *RELN*. The application of artificial neural networks was used to predict Autism Diagnostic Observation Schedule—Second Edition (ADOS-2) scores relative to environmental risk factors, such as high gestational weight, maternal age, preterm age, lack of folic acid intake, low birth weight, and living conditions, and showed that they are good predictors for ASD [94, 95]. In summary, investigating these environmental factors yielded crucial insights into epigenetic differences in genes, offering better intervention measures and even individualized therapeutic approaches.

#### Asia

Several environmental factors have been identified on the Asian continent as associated with both DNA methylation and autism or only with autism. These studies were from China, Saudi Arabia, Japan, South Korea, India, Lebanon, and Taiwan.

Most studies from China used a candidate gene approach and compared differences in methylation levels between ASD and controls. When comparing *ST8SIA2* gene methylation levels in children with ASD to those in controls, Yang *et al*. discovered that ASD children had greater methylation levels at Chr. 15: 92 984 625 and Chr. 15: 92 998 561 [96]. There was also a negative correlation between *ST8SIA2* expression levels and stereotypical behaviors in the ASD group and a positive correlation with daily life skills. Wang *et al*. focused on seeing differences in DNA methylation of CpG islands in the *ESR2* gene between ASD and neurotypical males [97]. Their results showed minimal overall differences in methylation between ASD and neurotypical males; however, they found that hypermethylation at eight specific CpG sites was linked to the severity of autism symptoms Hu *et al*. conducted an analysis of the promoter region of *HTR4* to assess for differences in methylation. They found significant decreases in *HTR4* methylation in males with ASD but no significant differences in females with ASD and no significant differences between neurotypical males and female subjects [98].

Additionally, other researchers investigated the potential connection between ASD and *APOE* methylation [99]. The study discovered that *APOE* methylation is considerably higher in pediatric patients with ASD than in controls, with a reference methylation percentage of 15.4% serving as the optimal predictor of ASD.

Zhao *et al*. investigated six apoptotic genes, *TGFB1, BAX, IGFBP3, PRKCB, PSEN2*, and *CCL2*, to determine whether any methylation changes were linked with ASD [100]. Hypomethylation of *TGFB1* was seen in peripheral blood samples of children with ASD, and there was a positive correlation between the Autism Behavior Checklist interaction ability score and *TGFB1* methylation. In another investigation, DNA methylation differences between manually selected spermatozoa (MSS) and zona pellucida-bound spermatozoa (ZPBS) were identified, and their association with ASD was examined [101]. MSS are sperm chosen based on visual assessment, while ZPBS are those that naturally adhere to the egg’s outer layer (zona pellucida). The global DNA methylation levels were much lower in the ZPBS than in the MSS. In ZPBS, hypo-methylation was detected in 52.3% of the 11 175 DMRs across the whole genome. These DMRs were associated with nearly half of the autism candidate genes. The authors concluded that the increased incidence of autism in offspring conceived with intracytoplasmic sperm injection might be due to variations in methylation levels between ZPBS and MSS. In a different study, Liang *et al*. used monozygotic twins to identify the role of DNA methylation in the development of ASD [102]. A total of 2397 differentially methylated genes in ASD blood were found by DNA methylation analysis. Differences in methylation of *SH2B1* were further verified by bisulfite pyrosequencing in the monozygotic twins with ASD that were concordant versus discordant and in a group of 30 pairs of sporadic ASD case-control. Compared to ASD-concordant monozygotic twins, those whose ASD was discordant had a more significant *SH2B1* methylation difference.

Two studies from Saudi Arabia were relevant to this review. Alshamrani *et al*. found that global DNA hypomethylation in peripheral blood neutrophils of children with ASD was associated with increased inflammation, characterized by elevated levels of inflammatory mediators such as CCR2 and MCP-1, alongside reduced *DNMT1* expression [103]. They hypothesized that the plasticizer Di(2-ethylhexyl) phthalate, a chemical commonly used to increase the flexibility and durability of plastics, downregulates *DNMT1* expression by inducing oxidative inflammation, contributing to the development of ASD. Using a candidate gene technique, Algothmi *et al*. assessed the level of DNA methylation at the transcription factor (*SP1*) binding site in the *ACSF3* promoter region [104]. The expression of *ACSF3* and *SP1* was correlated in patients with ASD despite the study’s inability to establish the significance of DNA methylation on the binding site of *SP1* inside the *ACSF3* promoter.

Japanese researchers used two machine-learning algorithms to identify a possible biomarker for adult high-functioning ASD [105]. The *PPP2R2C* gene, which has the methylation annotation cg20793532, was shown to be downregulated and hypermethylated in the blood of ASD patients compared to the control group. The area under the curve (AUC) value was 0.79, and pyrosequencing was used for validation.

The other epidemiology studies were from India, Lebanon, China, South Korea, and Taiwan. Although these will not be described in detail, they found factors that were either associated with ASD or neurodevelopment, including CO, NO_2_, PM10 [106], SO2, Pb [107], older parent’s age, male sex, unhappy maternal feelings during pregnancy, living close to industrial regions, previous childhood infection [108], excessive fetal movement, maternal respiratory infection, maternal vaginal infection, maternal hypothyroidism, and family history of neurodevelopmental disorders [109]. This overview of the Asian region reveals a complex link between environmental factors, DNA methylation, and ASD. It suggests that DNA methylation patterns could serve as biomarkers for ASD diagnosis, highlighting the need for further research to understand the etiology of ASD.

#### Oceania

New Zealand and Australian investigators have conducted most of the ASD epidemiology and DNA methylation research in the Oceania region. A study conducted in New Zealand by Noble *et al*. examined the DNA methylation patterns in children who were exposed to maternal tobacco smoking during pregnancy [110]. The research aimed to determine the relationship between these methylation patterns and the development of conduct disorder characteristics. They discovered substantial differential DNA methylation of CpG sites in *CYP1A1, ASH2L*, and *MEF2C* in those with conduct problems who had been exposed to smoke in utero. Although these genes are not directly associated with vulnerability to ASD, they are connected to neurodevelopment [111–113].

Further research, which comprised groups of individuals from New Zealand and the United Kingdom, examined the impact of exposure to cannabis during pregnancy on alterations in DNA methylation in genes related to neurodevelopment [114]. The research revealed significant differences in DNA methylation throughout the whole genome in people at ages 0, 7, 15–17, and 27, which were linked to exposure to cannabis during pregnancy, both on its own and in combination with tobacco. The genes *LZTS2, NPSR1, NT5E, CRP2, DOCK8, COQ5*, and *LPAR5* contained CpG sites that were differentially methylated and were shown to be shared across several periods. These are also essential genes for neurodevelopment, which have implications with ASD.

An epidemiological study from Australia that included 182 infants revealed that several factors were linked to an increased risk of ASD [115]. These factors included being male, being born preterm, having a mother aged 35 years or older, having a mother born outside Australia, and being part of multiple births. Some factors associated with ASD have been studied in different countries. Preterm birth was found to be associated with altered DNA methylation of *HYMAI, PLAGL1, ZNF217*, and *OXTR* implicated in neurodevelopment [116, 117]. This shows the necessity for further investigation in the field, as additional studies on DNA methylation can provide more insights into the regional environmental factors of this area.

#### Latin America and the Caribbean

The studies for environmental factors associated with ASD and DNA methylation from Latin America and the Caribbean that we gathered were from Brazil, Mexico, and Jamaica. The studies found in Mexico were both on DNA methylation and ASD. To discover ASD-associated alterations in DNA methylation, Aspra *et al*. carried out an epigenome-wide study in the buccal epithelium [118]. They discovered ASD-associated hypomethylation of DMRs linked to the *RASGRF2, GSTT1, FAIM*, and *SOX7* genes, as well as hypermethylation of DMRs linked to the *ZFP57, CPXM2*, and *NRIP2* genes. In the other research, 853 CpGs with differential methylation were found in individuals with ASD [119]. They also discovered 64 genes included in the SFARI gene database of ASD risk candidates. The genes *ISM1, PTPRG, SLITRK4, CAP2*, and *CYP26C1* included the five most statistically significant differentially methylated CpGs in ASD.

The impact of environmental variables on the clinical heterogeneity of ASD was investigated in a Brazilian study using the epigenetic clock and vulnerability components at birth as indicators [120]. The epigenetic clock, a biomarker of biological aging based on DNA methylation levels at specific CpG sites, allows researchers to estimate the biological age of tissues and cells. In this context, it was used to assess whether early-life environmental exposures could accelerate biological aging, thereby contributing to the observed clinical heterogeneity in ASD. Researchers discovered a high concentration of differentially methylated probes in CpG sites within variably methylated regions, influenced by environmental and genetic factors. The hypermethylated sites were associated with functional single nucleotide polymorphisms within gene regulatory regions, suggesting potential G×E interactions for common genetic variants in ASD.

Four epidemiological studies from Brazil examined the different perinatal and maternal factors related to ASD [121–124]. ASD was found to be associated with the following outcomes and conditions as reported by these studies: congenital malformation, neonatal jaundice, absence of crying at birth, childhood seizure episodes, gestational infection, gastrointestinal symptoms, obesity, obesity-related complications, meconium-stained amniotic fluid, cesarean section delivery, two or more adverse peripartum events, prematurity, low birth weight, and perinatal asphyxia.

Three studies in Jamaica examined various environmental variables and their association with ASD [125–127]. Christian *et al*. discovered that maternal exposure to fever or illness, physical trauma, and oil-based paints were associated with ASD [125]. Furthermore, the influence of maternal exposure to oil-based paints on the association between maternal exposure to pesticides and ASD in children may act as an effect modifier. The other two research studies investigated the effect of drinking water sources, vegetable and seafood diet, and blood arsenic and mercury contents in ASD patients. One study discovered that drinking water sources, eating avocado, and eating “callaloo, broccoli, or pok choi” were all connected with increased arsenic levels [126]. However, after controlling for other variables, they discovered no significant associations between blood arsenic levels and ASD. In the second investigation, children who ate seafood had higher blood mercury levels than children residing in the USA or Canada in both ASD cases and controls. Still, no association was observed between ASD and mercury levels after controlling for multiple factors [127]. Their results also revealed that children with parents who have a high school education were at a greater risk of mercury exposure than children with at least one parent with a higher level of education. The research from Latin America and the Caribbean, encompassing studies from Brazil, Mexico, and Jamaica, highlights the complex interplay between environmental factors, DNA methylation, and ASD. These findings contribute to the growing body of evidence suggesting that both genetic and environmental variables play critical roles in the development of ASD, underscoring the need for further investigation using DNA methylation across diverse populations and regions.

#### Africa

Several elements of the African continent have been recognized and examined. Malawi, Benin, and Tanzania all investigated and considered malaria as an environmental risk factor for ASD because it is more prevalent in African countries. However, those factors have only been examined about ASD or the prevalence of neurodevelopmental disorders rather than examining the DNA methylation in ASD biospecimens. With over 125 million pregnant women at risk of malarial infection, a few studies show that maternal infection during pregnancy without congenital infection was associated with an increased risk for neurocognitive defects in offspring [128]. In Benin, they performed a study measuring the prevalence of malaria infection before pregnancy and placental malaria, defined as the accumulation of plasmodium-infected red blood cells in the placenta [129, 130].

Additionally, regions in Africa at risk of malarial infection are controlled by indoor residual spraying with dichlorodiphenyltrichloroethane (DDT) and pyrethroids, and exposure to such chemicals is known to be associated with neurodevelopmental delay [131]. While studies suggest malarial infection as a potential risk factor, investigations of DNA methylation effects and gene expression analyses have not yet been performed to observe genetic pathways and regulation in response to malaria that may produce ASD-related phenotypes. Furthermore, studies in these regions contain significant environmental factors that may contribute to ASD, such as high rates of HIV, helminth infections, and significant economic and food insecurities [131]. Despite the limited resources and challenges, examining multiple variables is highly limited, and one can only observe the most prevalent factors within that country of research.

Similarly, research conducted in Egypt was limited to observing exposure to mercury, lead, and aluminum levels through hair analysis [132]. While there were no statistically significant relations between levels of mercury, lead, and aluminum and ASD severity, interestingly, elevated hair concentrations of heavy metals were observed in autistic children and correlated with the severity of symptoms [132]. For studies in Egypt, these studies did not examine DNA methylation and its relation to environmental variables and the phenotype. Interestingly, South Africa has been one of only a few African countries performing genetic molecular research, which can be improved with more availability of resources and funding. A study at the University of Cape Town looked at the DNA methylation of *PGC1α* and its associated genes, such as *STOML2, MFN2, FIS1, OPA1*, and *GABPA*, all related to mitochondrial regulation [133]. Within the South African cohort, *PGC1α* was hypermethylated in ASD samples and clustered around the transcriptional start site between the five prime untranslated regions (UTRs) and intron 1. In contrast, intron 2, 12, and 3 prime UTRs were hypomethylated.

One primary concern with all African studies is the sampling methods, especially with diagnosis. Different studies performed different diagnoses, mainly due to the lack of medical professionals who can perform such diagnoses. Furthermore, many economic or social demographic variables may influence DNA methylation, which can be a significant confounding factor that we cannot ignore. This may apply to other studies in different world regions, but this issue is most prominent in Africa.

## Discussion

### Prospects for prevalence studies in ASD worldwide

The first thing that can be appreciated is that although prevalence estimates have shown that there is an apparent recent increase in ASD, there is significant variability in the estimates, which makes it difficult to compare between studies. There are differences in the diagnostic criteria that are used. A universal diagnostic approach would help account for the heterogeneity between the studies. Also, in formulating a universal diagnostic tool, it will be essential to formulate one that is culturally relevant and appropriate. For instance, avoiding eye contact in some cultures is shunned, so it will be essential to consider that. Although some countries have translated the DSM-V and M-CHAT into their languages, more needs to be done [134–136]. The diagnostic criteria are challenging when the clinical definition of ASD changes, which is the case for DSM-IV and DSM-V [137].

Secondly, there might be an underestimation of prevalence estimates in some regions due to a lack of trained personnel, lack of resources for both the patients and healthcare facilities, social stigma that might exist about mental disorders, religious beliefs, and lack of awareness within communities [4, 35, 38, 138–143]. Promoting funding to less privileged communities is essential as it might help support those needing services.

Finally, prevalence data showed that males are diagnosed more often than females, which raises important questions about potential diagnostic biases and the underlying mechanisms of sex differences in ASD. Some researchers have mentioned that this bias toward diagnosing males rather than females might be due to sex-specific behavioral manifestations, with females having socially acceptable behaviors that might not meet the diagnostic criteria [4, 30, 142]. The other possibility is that environmental factors might interact differently in males and females, leading to differences in risk and disease manifestation [144]. These differences might also be explained by the female protective effect, in which females would need a higher genetic or environmental burden to present with ASD [145]. Therefore, differences in ASD prevalence estimates across different regions, along with sex differences in diagnosis, show the critical need for standardized, culturally sensitive diagnostic criteria and increased awareness to ensure all individuals with ASD, regardless of location or sex, are accurately identified and supported.

### Comparative analysis of findings within and across continents

We have summarized the results of research studies that examined DNA methylation at the interface of environmental risk factors for ASD across different continents and countries, as well as studies examining the environmental factors in countries where DNA methylation studies were lacking. Overall, the results of this comprehensive review point to areas of convergence between studies and significant gaps in research in this critical area.

#### Findings within continents

In North America and Europe, we focused on studies that showed how different environmental factors affect DNA methylation and how that is associated with ASD. Studies from these regions have identified specific environmental exposures, including cannabis use, air pollution, maternal smoking, and exposure to POPs, associated with DNA methylation changes in genes related to ASD. These findings underscore the importance of considering both genetic predispositions and environmental exposures in understanding ASD’s etiology. These continents’ research capacity and healthcare infrastructure have facilitated large-scale epidemiological and molecular studies, allowing for a more nuanced understanding of ASD. However, despite these advances, challenges still need to be addressed, particularly ensuring that findings are inclusive and representative of diverse populations. This region is pushing toward integrative approaches to ASD and constantly creating technological advancements. While innovative, it is also important to share common ground with other regions worldwide to be more inclusive by investigating the efficacy of such approaches worldwide.

Asia presents a varied landscape of ASD research, with studies highlighting different environmental factors—such as exposure to plasticizers, pesticides, and heavy metals—that may contribute to the disorder. The research from China emphasizes the role of candidate genes and their methylation status in ASD, suggesting potential biomarkers for the disorder. However, the continent faces challenges in standardizing diagnostic criteria and methodologies, which complicates efforts to fully understand ASD’s prevalence and etiology across diverse Asian populations. With cultural stigma toward neurodevelopmental diseases, the acceptance of treatment and recognition of ASD is severely limited. Indeed, it is essential to emphasize the importance of the unification of diagnostic criteria; it is also crucial to spread education and awareness that would allow the destigmatization of ASD in Asian countries.

In Oceania, particularly Australia and New Zealand, there is a notable recent increase in ASD prevalence, alongside research into environmental factors such as maternal smoking and cannabis exposure during pregnancy. These studies contribute to the growing body of evidence linking prenatal environmental exposures to changes in DNA methylation patterns associated with ASD. However, the region’s molecular research is still in its early stages, with a need for more comprehensive studies to explore the complex interplay of genetic, environmental, and epigenetic factors in ASD.

Research from Latin America and the Caribbean is limited. Still, it suggests that perinatal and maternal factors may play a role in ASD, with some findings also found in more extensive studies [146, 147]. The studies available highlight the potential for DNA methylation studies for ASD since they can be linked to the environmental factors they found. However, they also demonstrate significant gaps in research capacity and infrastructure that need to be addressed to understand ASD in these regions better. Collaborative funding and research toward investigating the prevalence of ASD must be a priority, as there are no accurate estimates compared to North America.

**Table 3.** Summary of key findings

Summary
**Convergent themes**
**Environmental exposures:** Several studies highlight common environmental risk factors such as maternal smoking, air pollution, heavy metals, and prenatal cannabis exposure that are associated with DNA methylation changes linked to ASD.
**Key Genes:** Consistent epigenetic changes are observed in *CYP2E1, DLGAP2*, and *OXTR* across multiple regions, suggesting their pivotal role in ASD etiology.
**Prenatal influences:** Prenatal exposures, including tobacco smoke, pesticides, and stress, are significant contributors to ASD, affecting DNA methylation patterns in key neurodevelopmental genes.
**Current gaps**
**Regional biases:** There are limited studies from Africa and Latin America, leading to potential biases in our understanding of ASD prevalence and etiology due to underdiagnosis and lack of resources in these regions.
**Diagnostic criteria:** Variability in diagnostic criteria and methodologies across studies complicates direct comparisons and the integration of findings from different regions. A standardized diagnostic approach is crucial.
**Genetic and environmental interactions:** More research is needed to understand the G×E interactions, particularly in genetically diverse populations, to uncover the complex mechanisms underlying ASD.
**Sample size and population differences:** Variations in sample sizes and population demographics across studies can influence the generalizability of the findings. Large-scale, diverse population studies are required.
**Proposed solutions**
**Standardization of diagnostic tools:** Implementing a universal diagnostic approach that is culturally relevant and appropriate to different regions can help standardize ASD diagnosis and improve comparability between studies.
**Enhancing research capacity:** Promoting funding and collaborations for research in underrepresented regions, particularly Africa and Latin America, can help address gaps in ASD prevalence and etiology data.
**Genome-wide discovery approaches:** Conducting genome-wide DNA methylation studies in diverse populations can ensure that findings are representative of the global population, accounting for differences in genetics, environments, and G×E interactions.
**Large-scale sequencing consortia:** Establishing large sequencing consortia for DNA methylomes like human genome sequencing projects can help overcome biases in current array-based platforms and improve the diversity of genomic databases.
**Advanced Technologies:** Utilize advanced sequencing technologies such as WGBS to overcome biases in current array-based methods and improve the comprehensiveness of DNA methylation studies.
**International Collaborations:** Fostering international collaborations can facilitate large-scale genomic and epigenomic studies, enabling data integration across different regions and enhancing the reproducibility and generalizability of findings.
**Cultural Sensitivity and Awareness:** Raising awareness and reducing cultural stigma toward ASD through education and media can improve acceptance and recognition of the disorder, facilitating early diagnosis and intervention.

Africa faces the most significant challenges in ASD research, with limited prevalence and molecular studies data. Some studies suggest that environmental factors like malaria due to immune activation may be relevant in some countries in this region. However, the lack of comprehensive molecular research shows the urgent need for increased research efforts to understand ASD’s unique manifestations and causes in African populations. The effects of malaria on DNA methylation at ASD-risk genes are worth further investigation. As global warming becomes more prevalent, vector-borne pathogens will likely become more prevalent in more geographical regions. Knowing more about the relevance of infectious diseases during pregnancy to ASD susceptibility and DNA methylation patterns will, therefore, be important in the future.

#### Common themes about specific genes and exposures in environmental epigenetic studies of ASD

Several environmental factors associated with DNA methylation changes at specific genes have been identified across different studies. These factors impact DNA methylation patterns and contribute to ASD risk. These factors have predominantly been identified during pregnancy, where they affect DNA methylation patterns in the offspring.

Air pollution, particularly exposure to NO_2_, O_3_, and PM_2.5_, has also been frequently linked to DNA methylation changes associated with ASD. Ladd-Acosta *et al*. reported that prenatal exposure to NO_2_ and O_3_ leads to methylation loss in *CYP2E1* [69], a gene that was also found in a methylation analysis of ASD in the placenta [65]. Further, studies by Wang *et al*. and Lee *et al*. demonstrated that exposure to CO, NO_2_, and Pb during pregnancy significantly increased the risk of ASD, indicating that air pollutants can induce epigenetic modifications in neurodevelopment-related genes [106, 107].

Maternal smoking during pregnancy is another common environmental factor associated with DNA methylation changes linked to ASD. Hannon *et al*. identified a significant association between maternal smoking and increased DNA methylation at specific loci, including *AHRR* [90]. Additionally, Rijlaarsdam *et al*. found that maternal smoking is associated with child autistic traits and changes in *OXTR* methylation [93]. These findings suggest that maternal smoking can impact the epigenetic regulation of neurodevelopmental genes, thereby increasing the susceptibility to ASD.

Heavy metal exposure, particularly to lead, cadmium, and manganese, has been implicated in altering DNA methylation patterns related to ASD. Aung *et al*. reported hypermethylation near genes such as *CYP24A1* in response to lead exposure [73]. Similarly, Mohamed *et al*. and Omotosho *et al*. found increased mercury, lead, and aluminum levels in autistic children, indicating that heavy metal exposure can disrupt neurodevelopment through epigenetic modifications [132, 148].

The impact of THC (cannabis) on DNA methylation and ASD risk has also been explored. Schrott *et al*. and Schrott *et al*. found that cannabis use is linked to hypomethylation in genes such as *DLGAP2* and significant alterations in methylation patterns in spermatogenic cells, affecting genes like *NR4A2* [81, 82]. These studies suggest that cannabis use during critical periods can influence the epigenetic landscape of neurodevelopmental genes, contributing to ASD risk.

Prenatal stress has been shown to induce DNA methylation changes associated with ASD. Rijlaarsdam *et al*. linked prenatal maternal stress exposure to child autistic traits and *OXTR* methylation [93], while Stoccoro *et al*. found that prenatal stress leads to aberrant methylation levels in genes related to neurodevelopment [95]. These findings highlight the role of prenatal stress in modulating epigenetic mechanisms that may influence ASD risk.

One limitation of attempting to summarize common themes is that all the exposures and at least half of the DNA methylation studies summarized in Table 1 resulted from testing specific hypotheses of candidate genes and/or exposures. Thus, the summary of these findings may be biased by ascertainment bias.

### Prospects for DNA methylation studies in ASD worldwide

While DNA methylation studies in ASD promise to yield a panel of methylated regions at specific gene loci that may predict risk for ASD with greater than 90% sensitivity and specificity, there are still many gaps to fill to achieve this goal. First, genome-wide discovery-based approaches should be performed in different global populations and countries to ensure a diversity of genetics, environments, and G×E interactions representative of ASD etiology. It is encouraging that some genes identified from EWAS were replicated across studies, including *CYP2E1, DLGAP2*, and *CSMD1*. Furthermore, some imprinted genes appear replicated in candidate and genome-wide studies. While most EWAS studies utilize the uniformity of Illumina Infinium array-based platforms, there is a concern about the bias of probe representation of these platforms. Infinium arrays are biased toward promoters and genic regions, which are overall enriched for lower genetic and epigenetic polymorphism compared to other areas of the genome. These arrays were also designed based on biased human genome maps of the past rather than the much more comprehensive current genome maps of human diversity across the globe. Therefore, sequencing-based discovery studies should be performed for DNA methylation in multiple countries across continents. Large sequencing consortia for DNA methylomes would be one way of solving these significant gaps, such as what has worked for human genome sequencing to improve the diversity of genomic databases. Furthermore, smaller funding mechanisms could promote global collaborations between researchers in underrepresented countries and those using cutting-edge genomic sequencing platforms.

A second significant gap for epigenetic and genetic research in ASD is the problems associated with variable ASD diagnosis across countries and within distinct populations within individual countries. The discovery of biomarkers depends on the quality of the subjects’ diagnoses in any study. A potential solution to this problem is for all countries to use the same diagnostic criteria through the established ADOS or other agreed-upon diagnostic tool. This is why the discovery of DNA methylation signatures of ASD may be best performed on human cohorts that have had a uniform diagnosis by trained professionals, including both ASD cases and controls. While such studies would be inherently smaller in sample size compared to those that take anyone based on parent-reported ASD diagnosis, they would yield reproducible results that are less biased by social determinants of ASD diagnoses. Ultimately, DNA methylation-based biomarkers hold the promise to provide a quantitative molecular assessment of risk for ASD at the interface of both genetic and environmental factors.

Finally, the concept of epigenetic aging provides valuable insights into the biological aging process and its potential role in ASD. DNA methylation-based measures, such as the epigenetic clock, allow researchers to estimate the biological age of tissues and cells, offering a novel avenue for understanding how early-life environmental exposures might accelerate aging processes in ASD. Accelerated epigenetic aging could contribute to the clinical heterogeneity observed in ASD, where individuals present with varying levels of symptom severity. Importantly, these measures may also serve as potential biomarkers for ASD, possibly predicting disease onset, progression, or treatment response.

## Conclusion

This study shows a worldwide view of ASD research with progress and gaps. While much research in North America and Europe has started to reveal the complex genetic and environmental interactions that exist in ASD, much remains unknown about ASD’s global prevalence and etiology. The variability in research focus, capability, and outcomes across continents signifies the importance of international collaboration and funding in ASD research, especially in areas with limited resources. Addressing these gaps will allow the global research community to gain a more comprehensive and inclusive understanding of ASD, allowing for better diagnosis and early intervention.
